# Astringent flavanol fires the locus-noradrenergic system, regulating neurobehavior and autonomic nerves

**DOI:** 10.1016/j.crfs.2025.101195

**Published:** 2025-09-11

**Authors:** Yasuyuki Fujii, Shu Taira, Keisuke Shinoda, Yuki Yamato, Kazuki Sakata, Orie Muta, Yuta Osada, Ashiyu Ono, Toshiya Matsushita, Mizuki Azumi, Hitomi Shikano, Keiko Abe, Vittorio Calabrese, Naomi Osakabe

**Affiliations:** a SIT Research Laboratories, Shibaura Institute of Technology, Minuma-ku, Saitama, 337-8570, Japan; b Faculty of Food and Agricultural Sciences, Fukushima University, Fukushima, 960-1296, Japan; c Functional Control Systems, Graduate School of Engineering and Science, Shibaura Institute of Technology, Minuma-ku, Saitama, 359-1144, Japan; d Department of Bio-science and Engineering, Shibaura Institute of Technology, Minuma-ku, Saitama, 337-8570, Japan; e Department of Applied Biological Chemistry, Graduate School of Agricultural and Life Sciences, The University of Tokyo, 113-8656, Japan; f Department of Biomedical and Biotechnological Sciences, University of Catania, 95124, Italy

**Keywords:** Astringency, Flavanol, Locus coeruleus -noradrenergic system, Stress response

## Abstract

Astringency is a characteristic exhibited by only a limited number of polyphenolic compounds which show high electrochemical activity and are susceptible to oxidation at neutral pH conditions like the oral cavity and small intestine. Large-scale intervention studies have demonstrated that the astringent flavanol (FLs) can restore hippocampal-dependent memory. However, due to the low bioavailability of FLs, the mechanism of action remains unclear. In this study, we aimed to elucidate the mechanism by which FLs acts on the nervous system through the gastrointestinal tract. Following a single gavage dose of FLs to mice, spontaneous motor activity in the open field and improved short-term memory in the novel object test were enhanced. Concurrently, activation of stress response systems, such as the sympathetic–adrenal–medullary axis (increased urinary catecholamines) and the hypothalamic–pituitary–adrenal axis (increased corticotropin-releasing hormone mRNA in paraventricular nucleus) was also observed. By the Mass imaging and *in situ* hybridization analyses, high-intensity noradrenaline (NA) originating from the locus coeruleus (LC) was revealed in the hypothalamus and brainstem immediately after FLs administration. These changes of NA have been suggested as the cause of enhanced memory, arousal and sympathetic activity. Furthermore, increased NA in the nucleus accumbens was observed as a response to visceral sensations induced by oral FLs administration. The present findings highlight how astringent stimulants FLs may activate brain function and the autonomic nervous system via gastrointestinal stimulation, causing physiological changes. These insights suggest that the sensory properties of food are important for maintaining homeostasis and promoting human health.

## Highlights

• A oral dose of astringent flavanols (FLs) enhanced spontaneous locomotion and short-term memory in mice.
• Flavanols was confirmed to enhance the stress response system.
• Flavanols immediately fired locus coeruleus-noradrenaline axis.
• Flavanol's astringency affects the brain and body, contributing to homeostasis.

## Graphical abstract

**Figure:**
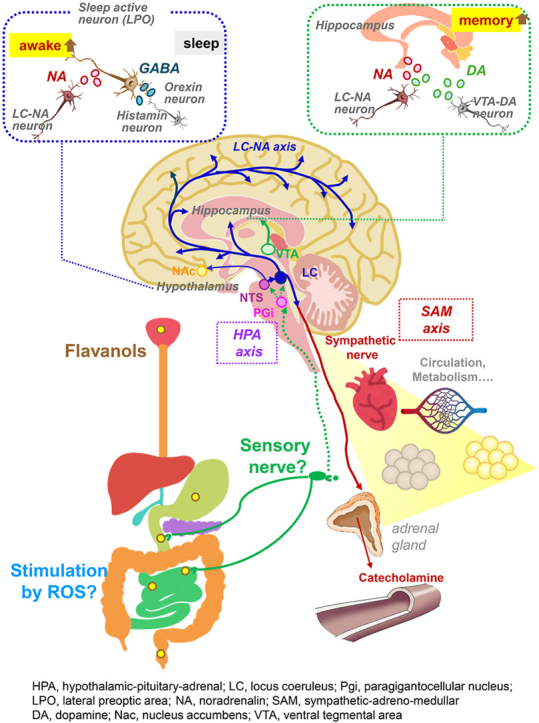

**Table:** Abbreviations

HPA	hypothalamic-pituitary-adrenal
LPO	lateral preoptic area
AD	adrenaline
CA	Catecholamine
CNS	central nervous system
CRH	corticotropin-releasing hormone
DI	discrimination index
D.W.	distilled water
DA	dopamine
DBH	dopamine β-hydroxylase
FLs	Flavanols
FMD	flow-mediated dilatation
ISH	*in situ* hybridization
LC	locus coeruleus
NA	Noradrenalin
NMET	Normetanephrine
NAc	nucleus accumbens
PGi	paragigantocellular nucleus
PVN	paraventricular nucleus
ROS	reactive oxygen species
SNS	sympathetic nervous systems
SAM	sympathetic-adreno-medullar
TRP	transient receptor potential
TH	tyrosine hydroxylase
VTA	ventral tegmental area
VMAT	vesicular monoamine transporter

## 1 Introduction

**Fig. 1:**
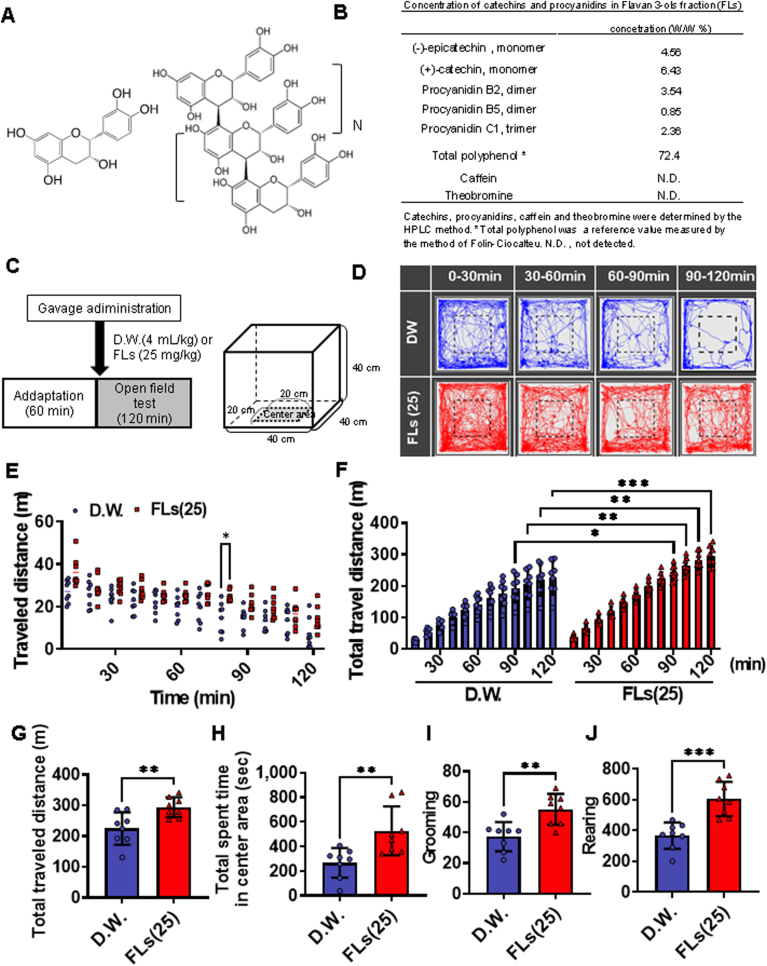
A single oral dose of flavanols (FLs) significantly increased mouse spontaneous activity. **A** chemical structure of flavanol; (−)epicatechin (left); procyanidins (right, n ≥ 0). **B** The composition of FLs. Catechins, procyanidins, and xanthine derivatives were measured according to the method of Natsume et al.(Natsume et al., 2000). **C** Scheme of the open field test. The open-field test was conducted following the administration of D.W. (blue, n = 8) or 25 mg/kg body weight flavanol (red, n = 8). **D** The typical subsequent behavioral trajectories of mice in an arena over 120 min. **E** Distance traveled by each mouse every 10 min. **F** Accumulated travel distance. **F** Total travel distance for 120 min. **G** The FLs-treated group significantly increased the total distance traveled compared with the D.W. group. **H** Duration spent in the center of the arena. **I** The number of grooming sessions. **J** The number of rearing sessions. The wakefulness indicators such as grooming and rearing were significantly increased in the FLs group compared to the DW group. To analyze multiple comparisons of travel distance or cumulative distance every 10 min, a two-way analysis of variance was performed, followed by Tukey's multiple comparison test. A Student's t-test was used to compare two groups of total travel distance, duration spent in the center of the arena, and the number of grooming and rearing sessions. The significance level is 0.05, with ∗ p < 0.05, ∗∗p < 0.01, and ∗∗∗p < 0.001.

Astringency, a stimulant property unique to polyphenols, is present in only a few compounds. Flavanols (FLs) and anthocyanin are typical astringent compounds (Osakabe and Terao, 2018). These compounds are characterized by their high electrochemical activity and susceptibility to oxidative degradation (Gioftsidi et al., 2022). In particular, under neutral pH conditions, such as in the oral cavity or small intestine, they are known to immediately produce reactive oxygen species and decompose to produce decomposition products or oxides condensed with decomposition products(Friedman and Jürgens, 2000; Miller et al., 2008; Xue et al., 2024). FLs are a subclass of plant flavonoids possessing a diphenylpropane structure and are categorized as monomeric (e.g., (+)-catechin or (−)-epicatechin) or oligomeric catechins (e.g., procyanidins) as shown in Fig. 1A (Osakabe and Terao, 2018). FLs are abundant in cocoa, red wine, and berries and closely related to the palatability of these foods.

According to a systematic review of previous studies, FLs may play a role in maintaining or improving cognitive function (Andrews et al., 2023; Zeli et al., 2022). Additionally, a recent large-scale intervention trial reported that one year of FLs intake restored hippocampal-dependent memory in older participants in the bottom third of habitual dietary quality with no habitual FLs intake (Brickman et al., 2023). Several studies have reported that within approximately an hour of ingesting FLs, there were significant changes in brain function (Socci et al., 2017), e.g., increasing mental tracking capacity (Massee et al., 2015; Scholey et al., 2010), mood (Pandolfi et al., 2016), attention (Karabay et al., 2018), and restored working memory performance(Grassi et al., 2016). A substantial number of animal studies have reported that FLs can inhibit neuronal cell death induced by apoptosis caused by neurotoxins such as oxygen radicals. This process can promote neuronal cell survival and synaptic plasticity (Yoon et al., 2025). Furthermore, it has been reported to promote angiogenesis, neurogenesis, and morphological changes in neurons within brain regions involved in learning and memory (Nehlig, 2013). The findings outlined above suggest that the ingestion of FLs is expected to maintain brain function through neuroprotective mechanisms.

These effects are hypothesized to be associated with the alteration of hemodynamics in brain (Decroix et al., 2019; Francis et al., 2006) and peripheral circulation (Hooper et al., 2012; Sun et al., 2019) immediately after FLs intake. Specifically, meta-analyses have demonstrated an increase in peripheral flow-mediated dilatation (FMD) 2 h after a dose of FLs (Sun et al., 2019). Our previous experimental animal studies found that this increase in blood flow after a single oral dose of FLs was due to hyperactivation of the sympathetic nervous systems (SNS) (Koizumi et al., 2021; Saito et al., 2016).

However, FLs are known to have very poor bioavailability (Osakabe et al., 2022; Osakabe and Terao, 2018). It is unlikely that orally ingested FLs are distributed in blood and directly affect the central or autonomic nervous system, therefore, the mechanism by which this change occurs remains unknown.

This study aimed to investigate the mechanisms by which FLs promote brain function. To achieve this, we observed spontaneous locomotion and cognitive function in mice following a single oral administration of FLs. Furthermore, the impact of FLs on both stress response systems, on the sympathetic-adreno-medullar (SAM) and hypothalamic-pituitary-adrenal (HPA) axis was assessed by urinary catecholamine (CA) excretion and expression of corticotropin-releasing hormone (CRH) mRNA in the paraventricular nucleus (PVN) of the hypothalamus. In addition, we observed the changes in dynamics in the whole brain for L-dopa, dopamine (DA), noradrenaline (NA), and the metabolite normetanephrine (NMET) using the MS imaging technique and with the mRNA expression of the synthetic enzymes and transporters of these neurotransmitters were determined to be used *in situ* hybridization (ISH).

## 2 Materials and methods

### 2.1 Materials

The FLs were prepared by the method we reported previously. Briefly, lipids were removed from the cacao liquor with n-hexane, followed by extraction with 80 % (v/v) ethanol. The extracts were passed through a chromatographic column (Diaion HP 2 MG; Mitsubishi, Kasei, Japan) and contaminants, including caffeine and theobromine, were removed by washing the column with 20 % (v/v) ethanol. A polyphenol fraction as FLs was eluted with 80 % ethanol, concentrated, and freeze-dried. Catechins, procyanidins, and xanthine derivatives were analyzed by high-performance liquid chromatography.

(HPLC) as described previously (Natsume et al., 2000). The HPLC apparatus used was a Tosoh instrument. FLs were prepared at a concentration of 0.1 mg/ml with 80 v/v % ethanol. This solution was applied to a SupelcosilTM LC-Si column (25 cm × 4.6 mm I.D., 5 mm). Elution was performed with the solvent system CH2CI2-MeOH-HCOOH-H2O (A) 5 : 43: 1:1 (v/v) and (B) 41 : 7:1 : 1 (v/v) using a linear gradient from 0 to 20 % A in 30 min, and from 20 to 100 % A in 10 min, followed by isocractic elution with 100 % A for 5 min. The composition of FLs used in the experiment was follows; (−)-epicatechin (monomer), 4.56 %(W/W); (+)-catechin (monomer), 6.43 %; procyanidin B2(dimer), 3.93 %; procyanidin C1 (trimer), 2.36 %; cinnamtannin A2 (tetramer), 1.45 %. Xanthine derivatives (caffeine, theobromine) were below the detection limit as shown in Fig. 1B.

### 2.2 Animals

Ten-weeks old male C57BL/6J mice were obtained from CLEA Japan, Inc. (Tokyo, Japan). For two weeks acclimation period, the animals were carefully handled to reduce anxiety behaviors. The study was conducted in accordance with the ARRIVE guidelines, and the protocol was approved by the Animal Experimentation Committee of Shibaura Institute of Technology (approval number: AEA 22007). As demonstrated in our previous research (Fujii et al., 2018), FLs have been shown to induce a stress response at doses of 10–50 mg/kg. Therefore, the dose used in the present experiment was set at 25 and/or 50 mg/kg. Mice used for MS imaging and ISH were decapitated and brains removed. All other mice were euthanized post-experiment with a triple anesthetic mixture (medetomidine, 0.75 mg/10 ml in physiological saline, Nippon Zenyaku Kogyo Co., Ltd., Fukushima, Japan; midazolam, 4 mg/10 ml in saline, Astellas Pharma Inc., Tokyo, Japan; butorphanol, 5 mg/10 ml in saline, Meiji Seika Pharma Co., Ltd., Tokyo, Japan) administered intraperitoneally in excess.

### 2.3 Open filed test for observation of spontaneous behavior

We observed the effects of a single oral dose of FLs on the spontaneous behavior of mice in the open-field test. Mice were gavage administrated with distilled water (D.W., n = 8), or FLs 25 mg/kg body weight (n = 8). The open fields are composed of four transparent acrylic chambers aligned horizontally or vertically. Mice were immediately placed in the center of an arena (40 x 40 × 40 cm), and their behavior was recorded for 120 min (Fig. 1C). The traveled distance was analyzed from the data, which were exported as a 30-min mp4 at 25 fps, corrected for tilt and brightness on filming using Premire Pro (Adobe Inc., California, USA), with a modification of the automatic tracking system developed by Zhang et al. (2020). All video files were processed by a MATLAB script running on a PC. The mouse activity code was designed to detect black mice such as C57BL/6J in a light arena.

### 2.4 Novel object recognition test

**Fig. 2:**
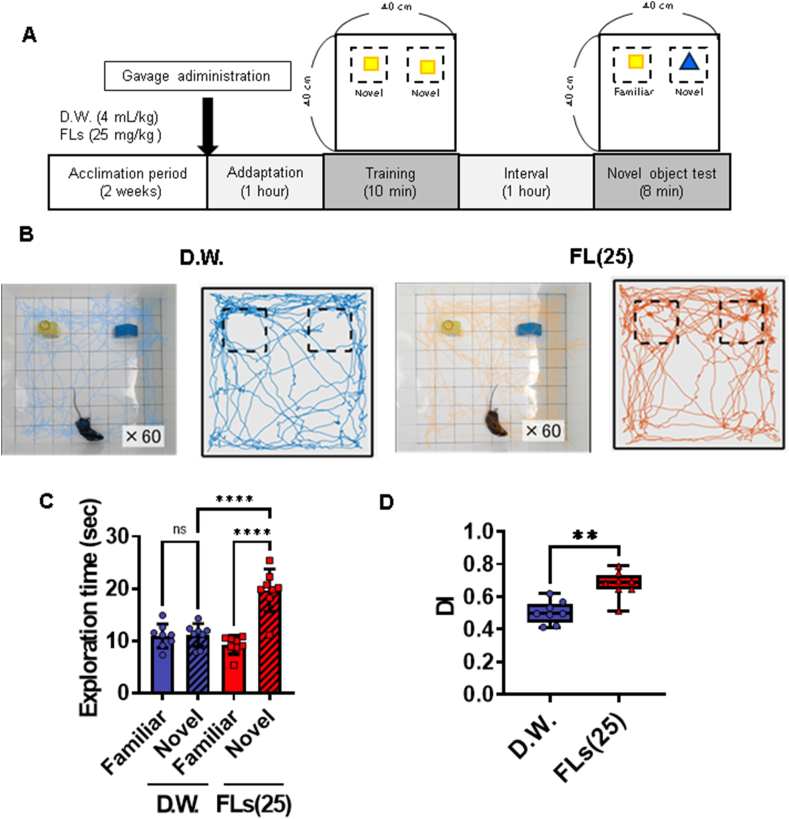
A single oral administration of flavanols (FLs) enhanced recognition memory in mice that was evaluated by a novel object test. **A** Scheme of the novel object test was conducted following the administration of D.W.(blue, n = 8) or 25 mg/kg body weight flavanol (red, n = 8) according to the method of Leger et al.(Leger et al., 2013). **B** The typical subsequent trajectories by mice during the 8 min following exposure to a novel object (right, D.W.; left, FLs 25 mg/kg). **C** Total exploration time for each group of objects. **D** The discrimination index (DI) results were expressed as a percentage of the total exploration time divided by the total exploration time to the novel object. To analyze multiple comparisons of exploration time, a two-way analysis of variance was performed, followed by Tukey's multiple comparison test. For comparisons between two groups of DI, the Mann-Whitney test was used. The significance level was 0.05, with ∗∗p < 0.01 and ∗∗∗∗p < 0.0001.

Novel object recognition test was performed according to the method of Ligar et al. with minor modifications as shown in Fig. 2A (Leger et al., 2013). After two weeks of acclimation, mice were divided into the D.W.-treated group and the FLs 25 mg/kg-treated group (n = 8 each). The experimental apparatus consisted of an acrylic arena (40 x 40 × 40 cm) with a transparent acrylic centre, into which a mouse was placed. The mouse was administered D.W. or FLs by gavage, and then allowed to acclimatise to the arena for 1 h. Two objects of identical appearance were positioned in opposite quadrants of the arena (i.e. the northwest and southeast corners), and the mice were permitted a period of 10 min during which to explore the objects freely. Subsequently, 1 h later, one of the objects was substituted for a novel object of a different shape and material. Thereafter, the mice were permitted to explore the arena for a period of 8 min in order to perform a novel object recognition test. The term “exploration” was defined as a behavior exhibited by mice whereby the mouse's noise is directed towards an object and within a distance of 2 cm from the object, with active vibrissae sweeping or sniffing. It should be noted that the duration spent in a seated position on the object, devoid of any indication of active exploration, was not incorporated within the aforementioned tally. The decision exploring or not exploring was analyzed from the data, which were exported as a 30-min mp4 at 25 fps, corrected for tilt and brightness on filming using Premire Pro (Adobe Inc., California, USA), with a modification of the automatic tracking system developed by Zhang et al. (2020). All video files are processed by a MATLAB script running on a PC. The mouse activity code was designed to detect black mice such as C57BL/6J in a light arena. To assess working memory, the discrimination index (DI) was calculated as the total exploration time to the novel object divided by the total exploration time, as previously described in a paper by Leger et al. (2013) as follows. [(time spent exploring novel object) − (time exploring familiar object)]/[(time spent exploring novel object) + (time exploring familiar object)].

### 2.5 Quantification of urinary CA excretion using HPLC

**Fig. 3:**
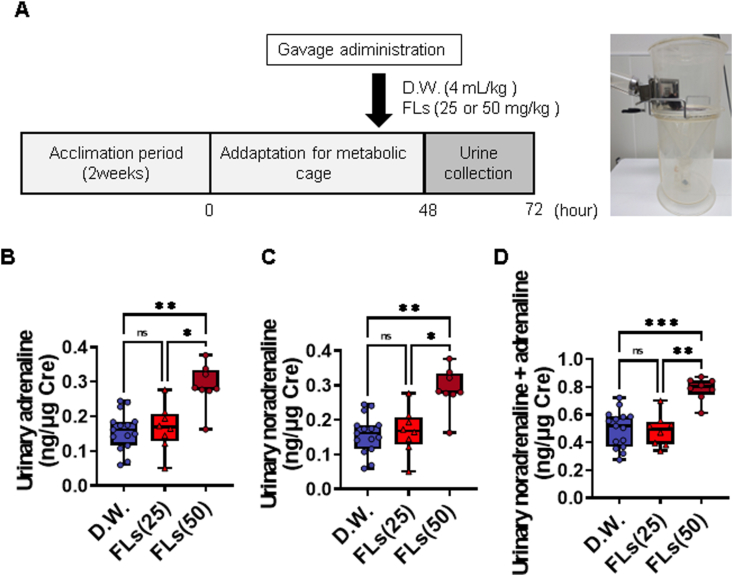
A single oral dose of flavanols (FLs) increased urinary excretion of CA. **A** Scheme for administration and urine collection protocol. After a 48-hrs acclimation period, 24-h urine samples were collected according to the method of Muta et al. (Muta et al., 2023). Urinary excretion of **B** noradrenaline (NA), **C** adrenaline (AD), and **D** total CA analysis in 24 hrs-urine (D.W. in blue, n = 15), 25 mg/kg (in light red) or 50 mg/kg (in dark red) flavanol (FLs, n = 8 pair each). CA excretion was expressed as a ratio to the urinary creatinine concentration. Statistical analysis for CA concentration was performed using the Kruskal-Wallis test followed by Dunn's multiple comparisons test. The significance level was 0.05, ∗p < 0.05, ∗∗p < 0.01 and ∗∗∗p < 0.001.

It has been suggested that brief periods of social isolation in metabolic cages can markedly alter SNS activity in mice (Takahashi, 2022). In our preceding research, we demonstrated that co-housing two mice in metabolic cages markedly reduced the stress response associated with single housing (Muta et al., 2023). Randomly assigned pairs of mice (n = 8 pairs each) were placed in metabolic cages and allowed to acclimate for 48 h. After that, the test chemicals were gavage administered, and urine samples were collected for 24 h (Fig. 3A). To prevent CA degradation, 20 μl of 2.5 mol/L HCl was added to the urine collection tubes. Oral administration of DW or FLs was performed between 10:00 and 11:00.

NA and adrenaline (AD) were purchased from Sigma–Aldrich. The urine was neutralized and heated for 10 min following incubation with 500 U/ml of the enzyme sulfatase from Helix pomatia Type H-2 (Sigma–Aldrich) for 1 h at 37 °C. Following this, isoprenaline (Sigma–Aldrich) was added as the internal standard, after which CA was purified using Monospin PBA® (GL Sciences, Tokyo, Japan). The HPLC system (Prominance HPLC System Shimadzu Corporation, Kyoto, Japan) comprised a quaternary pump with a vacuum degasser, a thermostatted column compartment, and an autosampler equipped with an electrochemical detector (ECD 700 S; Eicom Corporation, Kyoto, Japan). A reverse-phase column (Inertsil ODS-4, 250 × 3.0 mm ID, 5 μm; GL Sciences) was utilized, with the column temperature maintained at 35 °C. The HPLC mobile phase comprised 24 mM acetate-citrate buffer (pH 3.5, -CH3CN, 100/14.1 v/v). The mobile phase flow rate was 0.3 ml/min, with an injection volume of 20 μl. Eluents were detected and analyzed at 500 mV. The excretion of CA was expressed as a ratio with the urinary creatinine concentration, which was measured using Laboassay creatinine (FUJIFILM Wako Pure Chemical Corporation).

### 2.6 Observation of c-fos and CRH dynamics using ISH

**Fig. 4:**
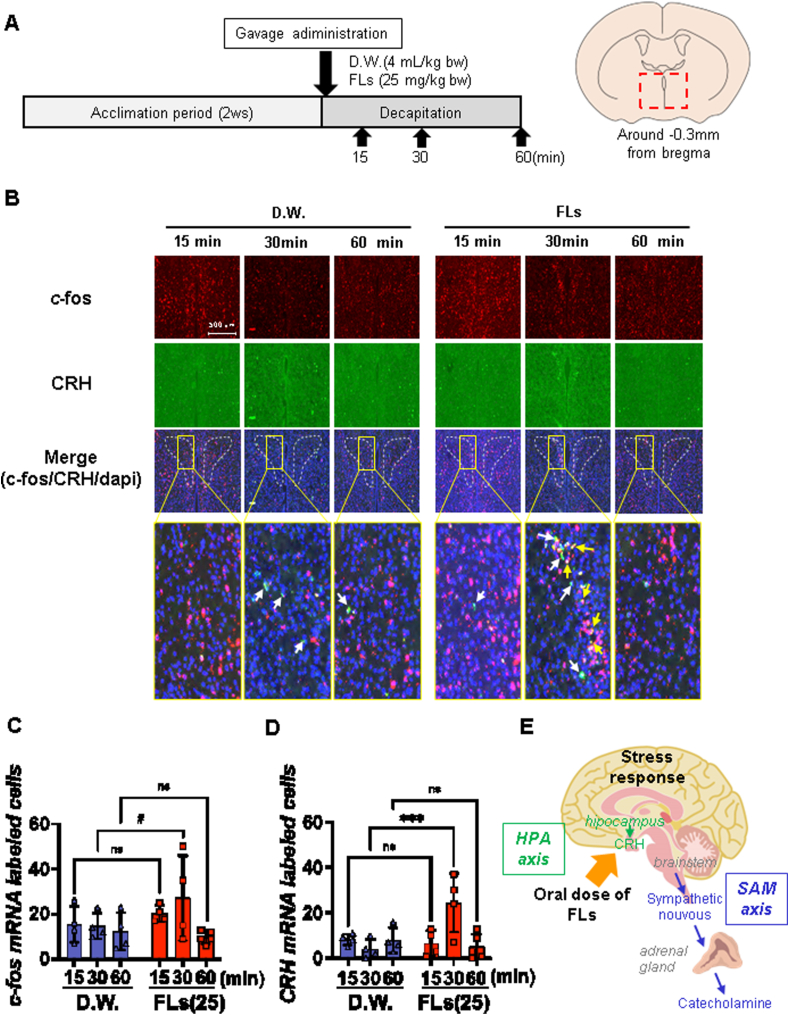
A single oral administration of flavanols (FLs) induced corticotropin-releasing hormone (CRH) mRNA in the paraventricular nucleus (PVN) of the hypothalamus. **A** Scheme of administration and brain collection protocol according to the method of Fujii et al. (Fujii et al., 2018). **B** A typical c-fos (top) and CRH mRNA (center) detected by ISH, as well as their merged and enlarged images (bottom, green as cfos; pink, CRH; blue, dapi). mRNA expression of cfos was demonstrated by the white arrow, and its CRH was shown by the yellow arrow in PVN. **C** The number of c-fos-positive cells, and **D** the number of CRH-positive cells in PVN enclosed by dotted line (n = 4 to 5). Compared to the D.W.-treated group, the number of CRH mRNA-expressing cells significantly increased in the FLs-treated group 30 min after administration. To analyze multiple comparisons of the number of positive cells, a two-way analysis of variance was performed, followed by Tukey's multiple comparison test. The significance level was 0.05, with ∗∗∗p < 0.001. **E** A diagram of a single oral dose of FLs enhanced the stress response system. SAM, sympathetic-adrenergic-medullary axis; HPA, hypothalamic-pituitary-adrenal axis.

The experimental protocol involved the gavage of mice with either D.W. (n = 4, each) or 25 mg/kg FLs (60 min after administration only, n = 5, 15 and 30 min after administration, n = 4). The mice were then decapitated 15, 30 or 60 min later (Fig. 4A). The excised brains were immediately frozen using liquid nitrogen and coronally sectioned (8 μm thick) using a cryostat (Leica, Wetzlar, Germany). The sections of fresh-frozen brains were prepared with a cryostat and thaw-mounted on glass slides (Matsunami Glass Ind. Ltd., Osaka, Japan). ISH was carried out to evaluate c-fos and CRH mRNA expression with the RNAscope® Multiprex Assay (Advanced Cell Diagnostics, CA, USA). An ImmEdge™ pen (H-4000, Vector Laboratories Inc., California, USA) was used to create a barrier around the sections. The barriers were dried completely at room temperature. The samples were treated with hydrogen peroxide (RNAscope® H_2_O_2_ and Protease Plus Reagents, Advanced Cell Diagnostics, CA, USA) for 10 min at room temperature, and washed twice in D.W. The antigens were activated with RNAscope® Target Retrieval Reagents (Advanced Cell Diagnostics) for 15 min, washed immediately in D.W., and dehydrated in ethanol for 1 min. The sections were treated with Protease Plus Reagents for 30 min at 40 °C in the HybEZ™ OVEN (Advanced Cell Diagnostics); then the sections were washed twice in D.W. Next, the RNA probe solution was mixed (1:50 dilution) with the target RNA probes: Mm-Fos (316921, Advanced Cell Diagnostics) and Mm-Crh-C2 (316091-C2, Advanced Cell Diagnostics). The sections were incubated with the probes for 2 h at 40 °C, then washed twice in wash buffer (Advanced Cell Diagnostics, CA, USA). Next, the sections were treated serially with AMP1 for 30 min (Advanced Cell Diagnostics), AMP2 for 15 min (Advanced Cell Diagnostics), AMP3 for 30 min (Advanced Cell Diagnostics), AMP4 for 15 min (Advanced Cell Diagnostics) at 40 °C, and AMP5 for 30 min (Advanced Cell Diagnostics), and AMP6 for 15 min (Advanced Cell Diagnostics) at room temperature. The sections were washed twice in the wash buffer for 2 min and treated with red conjugates (60:1), Fast red A and Fast red B, for 10 min at room temperature. After washing in wash buffer, the sections were treated successively with AMP7 for 15 min (Advanced Cell Diagnostics), AMP8 for 30 min (Advanced Cell Diagnostics) at 40 °C, and AMP9 for 30 min (Advanced Cell Diagnostics), and AMP10 for 15 min (Advanced Cell Diagnostics) at room temperature. Next, the sections were treated with green conjugates (50:1) as Fast green A and Fast green B (Advanced Cell Diagnostics), for 10 min at room temperature. The sections were rinsed twice in D.W. Finally, the sections were immersed in 50 % Gill hematoxylin (GHS116-500 ML, Sigma Aldrich, St-Louis, USA) for 30 s and washed twice immediately in water. After drying completely in a dry oven (MIR-162-PJ, Panasonic, Osaka, Japan) at 60 °C, the sections were covered with Vector mount™ permanent mounting medium (H-5000, Vector Laboratories Inc., California, USA).

### 2.7 Observation of noradrenaline dynamics using MS imaging and ISH

**Fig. 5:**
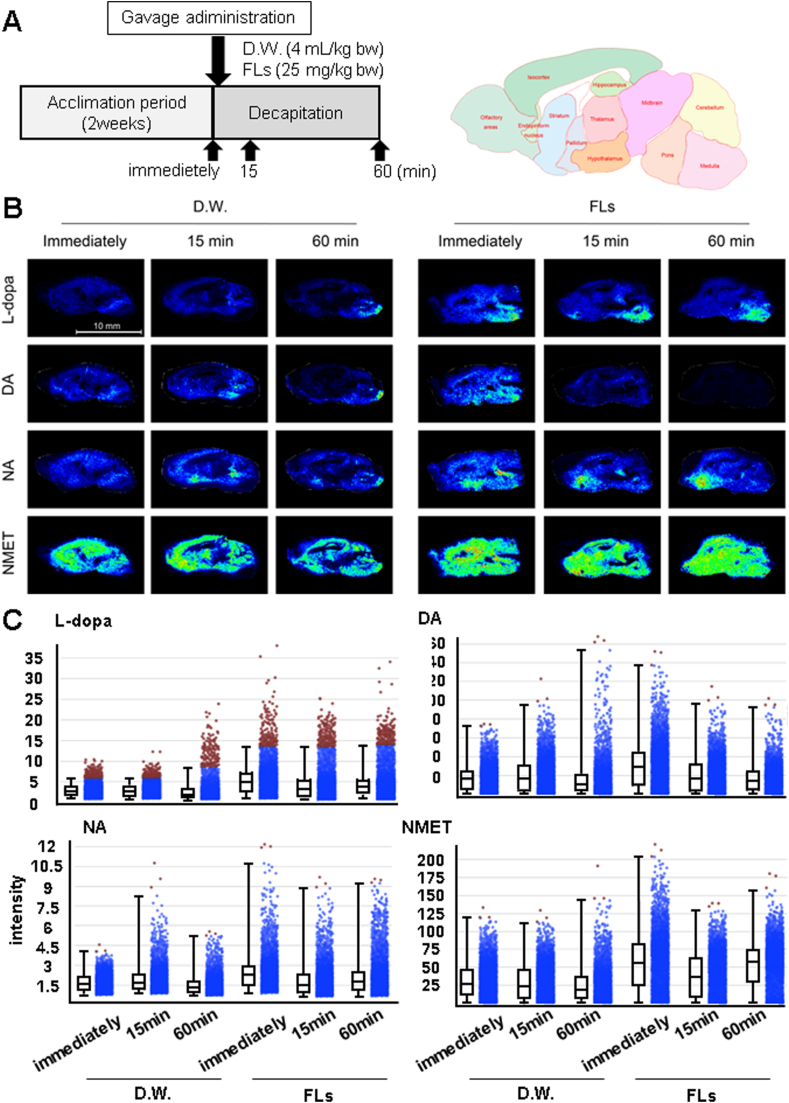
FLs caused substantial perturbations in L-dopa, dopamine, and noradrenaline FL and the metabolite noretinephrine (NMET) in the whole mouse brain. A Diagram of the dosing and brain harvesting protocol and a schematic of the mouse brain. Mice were given a single oral dose of D.W. or 25 mg/kg flavanols, brains harvested by decapitating immediately, and 15 or 60 min after administration. Excised brains were immediately frozen and sectioned (8 μm thick) using a cryostat. B Representative MS images of L-dopa (top), dopamine (DA, second from top), noradrenaline (NA, third from top), and normetanephrine (NMET, bottom). C Relative intensity of L-dopa, DA, NA, and NMET of the image B.

Mice were gavage administered with either D.W. or 25 mg/kg FLs and then decapitated immediately after, 15 or 60 min later (Fig. 5A). The study was conducted using a sample size of n = 2 for each group. The excised brains were immediately frozen using liquid nitrogen and sectioned (8 μm thick) using a cryostat (Leica, Wetzlar, Germany). The sections of fresh-frozen brains were prepared with a cryostat and thaw-mounted on conductive indium-tin-oxide-coated glass slides (SI0020M, Matsunami Glass Ind. Ltd., Osaka, Japan). A pyrylium-based derivatization method was applied for the tissue localization imaging of NA, its precursors L-dopa and DA, and its metabolite NMET according to the method by previous publication (Shikano et al., 2022). In brief, a solution of 2,4,6-trimethylpyrillium tetrafluoroborate (TMPy,4.8 mg/200 μL) (Taiyo Nippon Sanso Co., Tokyo, Japan) was applied to brain sections using an airbrush (Procon Boy FWA Platinum 0.2-mm caliber airbrush, Mr. Hobby, Tokyo, Japan). To enhance the reaction efficiency of TMPy on sections, the TMPy-sprayed sections were placed into a dedicated container and allowed to react at 60 °C for 10 min. The container contained two channels in the central partition, to wick moisture from the wet filter paper region to the sample section region. The filter paper was soaked with 1 mL methanol/water (70/30 vol/vol) and placed next to the section inside the container, which was then completely sealed to maintain humidity levels. The TMPy-labeled brain sections were sprayed with matrix (α-cyano-4-hydroxycinnamic acid-methanol/water/TFA = 70/29.9/0.1 vol/vol) using an automated pneumatic sprayer (TM-Sprayer, HTX Tech., Chapel Hill, NC, USA). Ten passes were sprayed according to the following conditions: flow rate, 120 μL/min; airflow, 10 psi; nozzle speed, 1100 mm/min. Each section was scanned to detect the laser spot area, and laser spot areas (200 shots) were detected with a spot-to-spot center distance of 120 μm using MALDI-Q-TOF MSI (rapifleX®, Bruker Daltonics Bremen, GmbH). The section surface was irradiated with yttrium aluminum garnet laser shots in the positive ion detection mode. The laser power was optimized to minimize the in-source decay of targets. Signals between m/z 200–800 were collected. Obtained mass spectrometry spectra were reconstructed to produce mass spectrometry images using Scils Lab software (Bruker Daltonics). Optical images of brain sections were obtained using a scanner (GT-X830, Epson, Tokyo, Japan) followed by MALDI-TOF MS of the sections. The detected masses of TMPy-labeled standard chemicals increased by 105.0 Da compared with the original mass, TMPy-l L-dopa, m/z 302.1; TMPy-l DA, m/z 258.1; TMPy-l NA, m/z 274.1; TMPy-l NMET, m/z 288.2. Tandem mass spectrometry confirmed the fragmentation ions of TMPy from the standard sample. A fragmented ion of the pyridine ring moiety was regularly cleaved and observed for all TMPy-modified target molecules.Tandem mass spectrometry confirmed the fragmentation ions of TMPy from the standard sample.

### 2.8 Fluorescence microscopy and quantification

All image analyses were performed by experimenters blinded to the experimental conditions. ISH image was taken using a fluorescence digital microscope (BZ-X800, KEYENCE Corp., Osaka, Japan). Cell counting of c-fos and CRH were performed throughout the extend of PVN. Each expression was quantified using NIH Image J software ver. 1.52 (http://rsb.info.nih.gov/ij/index.html).

### 2.9 Statistical analysis

The sample size was determined based on the results of a preliminary experiment, which utilized a power test with a significance level of 0.05 and a power of 0.9. All quantitative assessments were carried out in a blind manner. No data was excluded from the analysis. All data were expressed as mean ± standard deviation. All data were analyzed using GraphPad Prism 10.2.3 (San Diego, CA, USA). The normality of the sample distribution was tested using the Shapiro-Wilk test. Comparisons between the two groups were made using the Student's *t*-test or the Mann-Whitney test. For statistics on urinary CAs, the Kruskal-Wallis test was performed, followed by Dunn's multiple comparisons test. Otherwise, two-way ANOVA followed by Tukey's multiple comparisons test as a post hoc test was used for multiple comparisons. The significance levels were defined at #p < 0.1, ∗p < 0.05, ∗∗p < 0.01, ∗∗∗p < 0.001.

## 3 Results

### 3.1 Astringent FLs increased spontaneous activity

The 120 min open field results after intragastric administration of D.W. or 25 mg/kg FLs were shown in the line plot in Fig. 1D, and the distance traveled every 10 min in Fig. 1E. The cumulative distance traveled by the FLs-treated group was significantly longer than that of the DW-treated group, from 90 to 120 min after administration (Fig. 1F). The total distance traveled (Fig. 1G), time spent in the central area (Fig. 1H), number of grooming (Fig. 1I), and rearing sessions (Fig. 1J) were all significantly higher in the FLs group than in the D.W. group. All data used in the experiment are shown in SFig. 1.

### 3.2 Astringent FLs enhanced cognitive function in novel object recognition test

Fig. 2B showed the line plot results of mice in the novel object test after a single dose of D.W. (left) or FLs (right). Typical mouse behavior was also shown in the supplemental video (SVideo1 and 2). Fig. 2C displayed the total search time for the two objects, while Fig. 2D showed the Discrimination Index (DI). The FLs treatment group showed a significant increase in search time for the novel object (Fig. 2C), which led to a considerable rise in DI (Fig. 2D).

### 3.3 FLs increased urinary CA excretion by activating SAM axis

Fig. 3 illustrates the impact of a single dose of FLs on SNS activity by quantified urinary CA excretion over 24 h. The total urinary noradrenaline (Fig. 3B), adrenaline (Fig. 3C), and total CA (Fig. 3D) levels demonstrated a notable elevation in the 50 mg/kg FLs group, in comparison to the D.W. group, although this was not observed in the 25 mg/kg FLs group. All data used in the experiment are shown in SFig. 2.

### 3.4 FLs increased mRNA expression of c-fos and CRH in PVN by activating HPA axis

Fig. 4 shows the results of observing the mRNA expression of c-fos and the stress hormone CRH in the hypothalamic PVN using the ISH method. The upper part of Fig. 4B shows c-fos, the middle part shows CRH, and the lower part shows the merged images of c-fos, CRH, and dapi. The white or yellow arrow indicated cfos or CRH mRNA signal. The number of cells expressing c-fos (Fig. 4C) and CRH (Fig. 4D) in the PVN enclosed by the dotted line was counted using these images. There was a slight increase in the number of cfos-expressing cells between the D.W. group and the FLs group 30 min after FLs administration (Fig. 4C). CRH-expressing cell number showed a significant increase in expression 30 min after FLs treatment (Fig. 4D). The findings indicate that a single oral dose of FLs elicits a stress response and stimulates the SAM and HPA axes (Fig. 4E).

### 3.5 FLs exerted a substantial impact on the dynamics of noradrenaline in the whole mouse brain

Fig. 5B showed the distribution of NA (third from the top), its precursor 3-hydroxy-L-tyrosine (L-dopa, top) and DA (second from top), and its metabolite NMET (bottom), detected in sagittal sections of mouse brain immediately after, 15 or 60 min after a single dose of D.W. (left) or 25 mg/kg FLs (right). L-dopa and NMET were detected at higher levels in the FLs group than D.W. group throughout the observation period. A higher intensity of DA was observed in the hypothalamus, midbrain, pons, and medulla immediately after administration of FLs, and was rarely detectable 15 or 60 min after administration. It was also observed a higher level of NA in parts of the hypothalamus, pons, and medulla immediately after administration of FLs. In addition, a greater NA was observed in the ventral striatum 15 or 60 min after administration of FLs. The respective intensities of L-dopa, DA, NA, and NMET were shown in Fig. 5C after administration of D.W. or FLs. The intensity of L-dopa and NMET was higher in the whole brain throughout the observation period by a single oral dose of FLs. DA intensity was greater immediately after the administration of FLs. Immediately or 60 min after a dose of FLs, the intensity of NA was elevated. All data used in the experiment are shown in SFig. 3.

### 3.6 FLs substantially increased noradrenaline intensity within LC, lateral preoptic area (LPO), and nucleus accumbens (NAc)

**Fig. 6:**
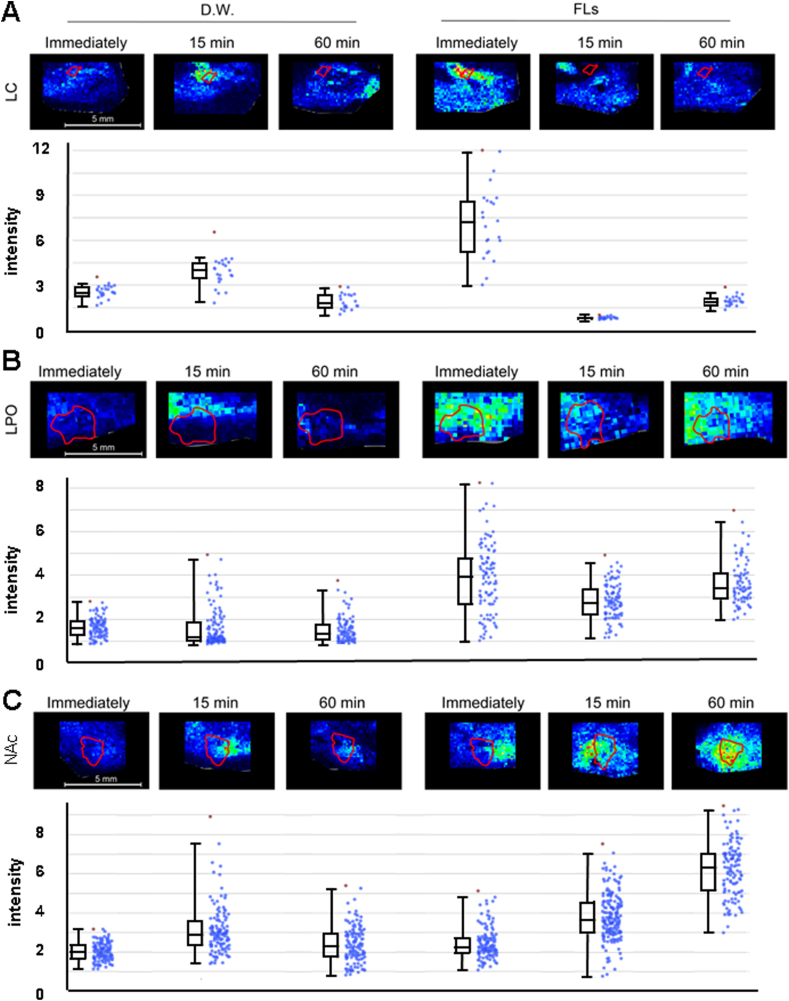
A single oral administration of flavanols (FLs) immediately enhanced the intensity of noradrenaline (NA) in locus coeruleus (LC), lateral preoptic area (LPO) and nucleus accumbens (NAc). **A** Representative MS images and relative intensity of NA in LC. **B** Representative MS images and relative intensity of NA in LPO. **C** A Representative MS images and relative intensity of NA in NAc.

In Fig. 6, the distribution and relative intensity of NA in LC, LPO and NAc. Fig. 6A shows the change in NA in LC after administration of FLs. Immediately after the administration of FLs, the intensity of NA in the LC markedly increased. Fig. 6B showed the change in NA in LPO after administration of FLs. The NA in LPO observed higher intensity at all durations in the FLs-administered group compared to the D.W.-treatment group. In Fig. 6C, NA signals in NAc following treatment of D.W. or FLs. The intensity of NA increased over time with FL administration. All data used in the experiment are shown in SFig. 4.

### 3.7 FLs resulted in an augmentation of noradrenaline biosynthesis or transporter production enzymes within the midbrain

**Fig. 7:**
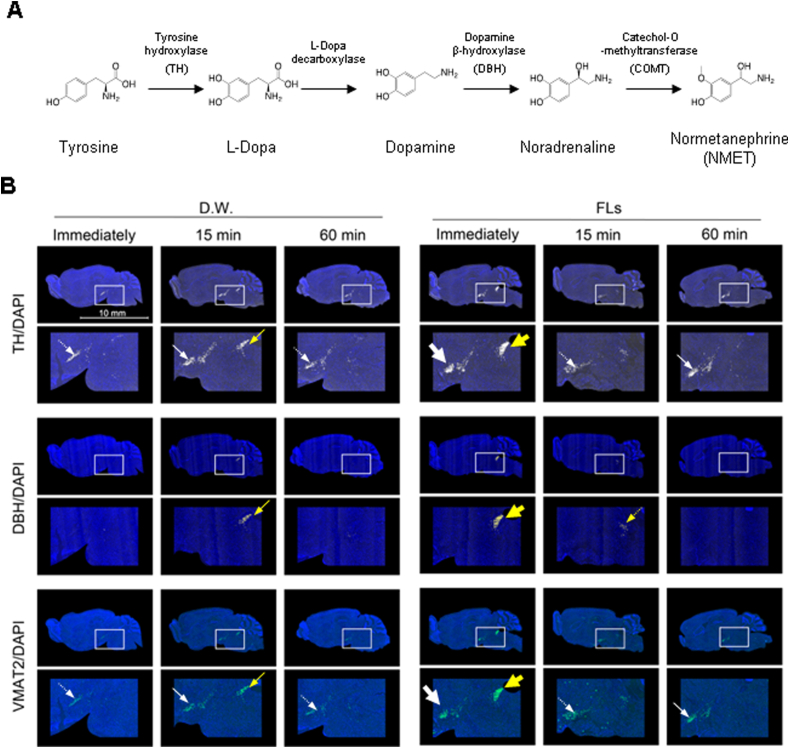
A single oral administration of flavanols (FLs) induced mRNA of synthesis enzyme and transporter for noradrenaline (NA) or dopamine (DA) in locus coeruleus (LC) or ventral tegmental area (VTA). **A** Pathways for the metabolism of DA and NA. **B** The mRNA expression of tyrsine hydroxylase (TH, top), dopamine β-hydroxylase (DBH, second from top), and vesicular monoamine transporter 2 (VMAT2, bottom) was assessed by using the ISH method. The expression in the LC was demonstrated by yellow arrows, and the expression in the VTA was shown by white arrows.

Given that a single oral administration of FLs resulted in high localized NA intensity, we analyzed the expression of enzymes involved in noradrenaline biosynthesis (Fig. 7A) or transporter production. Fig. 7B shows the mRNA expression of each enzyme and its enlarged image in frozen brain serial sections obtained immediately after FLs administration, and 15 and 60 min later using the same method as in Fig. 6. From the top to the bottom, the biosynthetic pathways (Fig. 7A), tyrosine hydroxylase (TH), dopamine β-hydroxylase (DBH), as well as a transporter, vesicular monoamine transporter (VMAT)2 were shown merged with dapi. In the FL group, as shown by the yellow arrows, a greater increase in TH mRNA expression was observed in the LC (yellow arrow) and ventral tegmental area (VTA, white arrow) immediately after administration, but it was attenuated after 15 or 60 min. On the other hand, in the D.W. group, it was only slightly detected 15 min after administration. DBH mRNA expression was observed as a strong signal in the LC (yellow) immediately after FLs administration. On the other hand, in the D.W. group, it was only slightly detected 15 min after administration. In addition, VMAT2 was marked increased in the LC (yellow) and VTA (white) immediately after administration in the FL group, and only slightly observed 15 min after administration in the D.W. group. All data used in the experiment are shown in SFig. 6.

## 4 Discussion

The series of experiments revealed that a single oral administration of FLs had a marked impact on neurotransmitter dynamics throughout the brain. In particular, the substantial increase in NA in LC and the induction of mRNA expression of the synthesizing enzyme and transporter in LC indicate the activation of the LC- noradrenergic neuronal network by a single gavage administration of FLs (Fig. 5, Fig. 6, Fig. 7). LC is the source of NA in the forebrain and is projected throughout the forebrain, brainstem, cerebellum, and spinal cord (Breton-Provencher et al., 2021). Furthermore, most cortical and subcortical regions are known to be under the control of LC-NA axonal nerves (Nomura et al., 2014; Zerbi et al., 2019).

The present study demonstrated that MS imaging revealed a significant increase in NA, not only in the LC, but also in the hypothalamic LPO, immediately after FL administration (Fig. 6A). Furthermore, the FL-treated group showed a significant increase in spontaneous motor activity and grooming/standing-up behavior, indicators of wakefulness, compared to the DW-treated group (Fig. 1). The relationship of these studies can be considered as follows; It is established that the release of NA from LC neurons plays an important role in regulating wakefulness. Specifically, optogenetic techniques have revealed frequency-dependent correlations between local neuronal firing, cortical activity, sleep-to-wake transition, and general motor arousal in the LC of the mouse (Carter et al., 2010). Conversely, it is widely acknowledged that LC-NA activity diminishes during sleep, but LC spikes generated by sensory input, such as sound stimulation, promote the transition to wakefulness as outlined below (Hayat et al., 2020). Released NA from LC activates β and α1 adrenaline receptors located within several subcortical structures, including LPO (Berridge, 2008). The LPO is considered the primary sleep-promoting nucleus in the brain, containing two major cell populations: excitatory glutamatergic neurons and inhibitory GABAergic neurons (Taksokhan and Kim, 2023). During the sleep cycle, glutamatergic neurons within the LPO become inactive, while GABAergic neurons become active, suppressing stimulation from NA, an arousal-promoting substance from the LC. Conversely, NA projecting to the LPO from LC spikes induces arousal by inhibiting GABA neuron activity, forming a “flip-flop” switch (Gallopin et al., 2000, 2005; Sangare et al., 2016). Based on the MS imaging results in Fig. 6A, gavage administration of FLs induced LC spikes, followed by NA projections from the LC to the LPO. Consequently, as shown in Fig. 1, the mice in FLs group maintained wakefulness, exhibiting higher levels of spontaneous activity and wakefulness indices compared to the D.W. group.

LC-NA neurons have been shown to regulate sleep, as well as to activate responses to novel stimuli and to enhance attention.(Sara, 2009; Sara and Bouret, 2012). Evidence from experimental studies on rodents indicates that pharmacological activation of the LC-NA has the potential to enhance memory through heightened attentiveness (Devauges and Sara, 1990). For instance, the depletion of monoamines in the LC, the antagonism of NA and DA in multiple brain regions, and the direct inhibition of the LC have all been demonstrated to impair memory across various tasks(Giustino and Maren, 2018; Lisman and Grace, 2005; Uematsu et al., 2017; Wagatsuma et al., 2018).In addition, the activation of the LC and the administration of DA and NA agonists to the LC have a positive effect on memory (Kempadoo et al., 2016; Packard and White, 1989, 1991).It is widely accepted that episodic-like memories formed in the hippocampus are retained long-term through a memory stabilization process termed early memory consolidation (Takeuchi et al., 2016; Yamasaki and Takeuchi, 2017). During the initial phases of memory consolidation, monoamine release from the LC to the hippocampus has been documented to facilitate the encoding of environmental stimuli and enhance memory retention over a timeframe of approximately 1 h. This process has been considered critically dependent on NA, which is secreted from the locus coeruleus, activating β-adrenergic receptors in the hippocampus (Takeuchi et al., 2016). In this study, we observed the effects of a single oral dose of FL on cognition using a novel object test. Mice that underwent memory training 1 h after FL administration demonstrated a significant improvement in novel object exploration time and a significant increase in DI. These results suggest that the stimulation of intragastric administration of FLs enhances the LC-dependent initial memory consolidation process (Fig. 2).

Moreover, LC has also been shown to release dopamine to the hippocampus and other cortical regions (Gálvez-Márquez et al., 2022; Kempadoo et al., 2016). In this experiment, FL administration did not detect DA or NA projections from the LC to the hippocampus by the method of MS imaging. However, ISH analysis revealed that FL administration immediately induced the expression of the DA-producing enzyme TH and the monoamine transporter VMAT2 in the LC (Fig. 7). FL administration also tended to increase TH and VMAT2 mRNA expression in the VTA. In response to significant events, dopamine neurons in the VTA are known to fire and innervate the pyramidal layer of the dorsal hippocampal CA1, thereby consolidating long-term memory (Sayegh et al., 2024). Therefore, these results suggest that FL stimulates NA and DA production in the LC and VTA, which then project to the hippocampus to consolidate short- and long-term memory. This may be one of the mechanisms underlying the improvement of hippocampal-dependent cognitive function in elderly people observed in large-scale, long-term intervention studies of FLs(Brickman et al., 2023). However, further research is needed to substantiate this hypothesis.

Previous studies have demonstrated that when healthy subjects consume foods rich in FL, FMD levels indicating increased peripheral blood flow significantly rise after 2 h (Hooper et al., 2012; Sun et al., 2019). However, as discussed later, the mechanism remained unclear due to the remarkably low bioavailability of FLs. To replicate this alteration, we established a skeletal muscle blood flow observation and evaluation system using rodents (Fushimi et al., 2021). Using this method, we observed a significant increase in skeletal muscle blood flow after intragastric administration of FLs, yielding results consistent with the intervention study findings. Conversely, co-administration of an adrenergic receptor antagonist abolished this change (Saito et al., 2016). These findings indicate that the observed increase in blood flow following FLs gastric administration results from SNS activation—specifically, NA released from sympathetic nerve terminals activates adrenergic receptors expressed on cardiac muscle and vascular smooth muscle. CA is not only released from nerve terminals but is also secreted into the bloodstream from the adrenal medulla in response to significant increases in SNS activity, such as during stress (Osakabe et al., 2023). Since most CA secreted into the bloodstream is conjugated and excreted in urine, it serves as a stress indicator (Muta et al., 2023). In this study, as shown in Fig. 3A, urinary CA concentration increased significantly in a dose-dependent manner. This result confirms that a single intra-gastric administration of FLs activates the SNS.

Generally, increased SNS activity is one of the physiological stress responses, and it is well known that the HPA axis is simultaneously activated. Classically, CRH is recognized as the master regulator of the HPA axis. It is known that stress exposure causes CRH to be secreted from the paraventricular nucleus of the hypothalamus to the pituitary gland and release of ACTH, promoting the secretion of glucocorticoids from the adrenal cortex into the bloodstream. Glucocorticoids form a negative feedback mechanism by suppressing CRH production via receptors expressed centrally, thereby inducing adaptation to stress (Osakabe et al., 2024). Recent studies have demonstrated that stress stimuli cause CRH release from afferent neurons in the hypothalamic paraventricular nucleus and also secret into the LC region, where CRH receptor 1 is expressed (Aston-Jones et al., 1991; Hauger et al., 2006; Valentino et al., 1983). This revealed a regulation mechanism whereby depolarization of LC neurons is induced, leading to the release of NA from axon terminals throughout the entire nerve axis (McCall et al., 2015; Ross and Van Bockstaele, 2020). This experiment confirmed that FL gastric administration stimulation significantly increased CRH mRNA expression in the paraventricular nucleus of the hypothalamus, as shown in Fig. 2B and c. Based on the above, it was considered that FLs stimulation acts as a stressor, activating the SNS and HPA axis.

On the other hand, the bioavailability of orally ingested FLs has been demonstrated to be exceedingly low. It has been established that some of the monomer (−)-epicatechin is conjugated immediately after absorption from the intestinal tract, and that blood concentrations peak approximately 30 min after ingestion (Osakabe et al., 2023; Osakabe and Terao, 2018). However, it should be noted that its absorption rate is only a few percent. Procyanidins, which are oligomers, are known to be absorbed only minimally, which makes detection in the blood difficult (Osakabe et al., 2023; Osakabe and Terao, 2018). Conversely, experiments employing epicatechin isotopes have documented 78 % absorption; however, a significant proportion of this is hypothesized to be products that have undergone decomposition within the digestive tract or metabolites by intestinal microbiota (Borges et al., 2016). Therefore, recent research has concentrated on alterations in the intestinal microbiota and the secondary metabolites produced in the colon following repeated administration of FLs (Corrêa et al., 2019; Wang et al., 2022). Specifically, 5-(3′,4′-dihydroxyphenyl)-γ-valerolactone is known as a characteristic compound detected after FLs intake, and a correlation has been observed between its urinary excretion amount and FLs intake levels (Yoon et al., 2025). However, there is currently a paucity of evidence to support the hypothesis that alterations in metabolite composition, associated with alterations in the gut microbiota, are directly linked to biological activity. In particular, changes in brain function and the circulatory system observed immediately after FL administration are expected to have a low correlation with changes in the gastrointestinal environment following long-term intake. Conversely, as the present study demonstrates, a single oral dose of FLs has been observed to alter neurotransmitters in the brain, induce stress responses, and significantly modify mouse behavior immediately. It is imperative to explore alternative mechanisms that do not involve the gut microbiome or metabolites.

FLs are a group of substances that have a potent astringent taste and have an essential influence on the sensory properties of chocolate and red wine, affecting their palatability (Ferrer-Gallego et al., 2015; Soares et al., 2020). Astringency is known to polyphenol-specific perception, but the mechanism by which mammals recognize astringent stimuli is currently unknown. Astringency, like other stressors such as capsaicin, may be perceived as a somatosensory (Mouritsen, 2016; Schöbel et al., 2014). According to the previous study, the sensation of astringency remained intact when the gustatory nerve was blocked by local anesthesia, but was eliminated when both the trigeminal and gustatory nerves were blocked (Schöbel et al., 2014). This finding suggests that the sensation of convergence is a somatosensory stimulus. It has been investigated that those somatosensory stimuli are input via the paragigantocellular nucleus (PGi), which is located in the anterior medulla oblongata, strongly activate the LC and NTS, resulting in the secretion of NA to NAc (Aston-Jones et al., 1991; Kerfoot and Williams, 2011). In the present results, we observed a rise in the NA in the NAc over time following an oral administration of FLs (Fig. 6C). In light of these findings, it can be reasonably hypothesized that the NA observed in the NAc after FL administration indicates the information transmitted via the NTS as a consequence of stimulation in the gastrointestinal tract.

Astringency of polyphenols including FLs was previously hypothesized to be caused by oral friction caused by the interaction of salivary proline-rich proteins and polyphenols which were detected by mechanoreceptors(Ferrer-Gallego et al., 2015). Conversely, recent studies indicate that salivary proteins may not be essential for perceiving astringency. Recently, it has been proposed that astringency is caused by the activation of transient receptor potential (TRPs) expressed in sensory neurons that sense pungency and reactive oxygen species (ROS). (Kurogi et al., 2015; Takahashi et al., 2021). For instance, ROS react with cysteine residues in the ankyrin repeats of TRP channels, causing sensory neurons to fire and transmit stimuli to the central nervous system (CNS) (Kozai et al., 2014). Polyphenols such as flavonoids and anthocyanins, which impart astringency, possess physicochemical characteristics including low stability and susceptibility to oxidative degradation and produce ROS, low-molecular-weight decomposition products, and high-molecular-weight oxidation products formed by the condensation of decomposition products, especially at neutral pH like oral cavity and intestine. (Friedman and Jürgens, 2000; Miller et al., 2008; Xue et al., 2024).In our previous study, the increase in skeletal muscle blood flow via hyperactivation of the SNS observed immediately after administration of epicatechin tetramer, a type of flavanol, to rodents was diminished by co-administration of the antioxidant N-acetylcysteine. In addition, this hemodynamic alteration of FL was marked suppressed by co-administration of a TRP-vanilid 1 or TRP-ankirin 1 antagonist (Fushimi et al., 2023). Based on these findings, it is undeniable that ROS produced by certain polyphenols in the gut are perceived as astringency and that this stimulation enhances neurobehavioural and sympathetically mediated circulation and metabolism. Further research is needed to explain how the somatic sensation of astringency is expressed through receptors such as TRP channels.

In the present experiment, alterations in neurotransmitters within the brain were observed following the ingestion of FL. However, several aspects require further elucidation. A notable challenge pertains to the elucidation of the mechanism through which astringent taste is perceived within the oral cavity and digestive tract. Amongst polyphenols, a limited number of compounds that yield astringent stimulation are recognized, and it is hypothesized that the perception of astringent taste is contingent on its chemical structure (Osakabe et al., 2024). As previously mentioned, it is imperative to substantiate the “astringent active oxygen hypothesis” proposing that astringent polyphenols generate ROS and activate TRP channels expressed on gastrointestinal sensory nerves. This can be demonstrated through the utilization of TRP channel knockout mice or electrochemically inactive astringent polyphenol derivatives. Additionally, it was observed that LC was promptly activated following the intragastric administration of astringent FL. However, the mechanism through which it affects other brain regions remains to be elucidated. In addition to identifying the areas activated by fMRI analysis following astringent polyphenol administration, it is essential to visualize the connectivity of each neuron using connectome technology to clarify spatial and temporal changes.

## 5 Conclusion

**Fig. 8:**
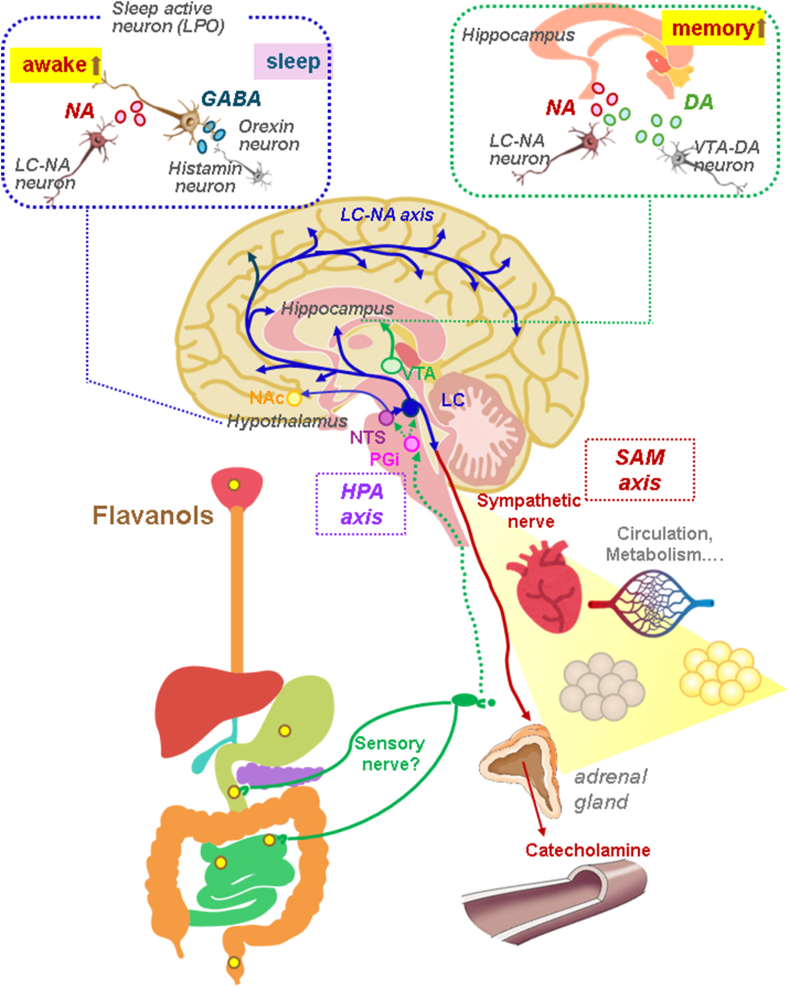
The impact of a single oral administration of flavanols (FLs) on the nervous systems. The oral administration of astringent flavanols (FLs) promptly transmitted a stimulus to the central nervous system, activating the hypothalamic CRH neurons, which subsequently secrete CRH to the locus coeruleus (LC), thereby activating the noradrenaline (NA) neural network. The projection of noradrenaline (NA) from LC to the hypothalamus preoptic area (LPO) has been observed to suppress sleep and promote wakefulness. The projection of NA and dopamine (DA) from LC and DA from the ventral tegmental area (VTA) to the hippocampus was suggested to enhance memory. The projection of NA from LC to the brainstem has been observed to activate sympathetic nerve activity, thereby augmenting circulation and metabolism.

In conclusion, the results reveal that a single oral dose of astringency FLs promptly activates LC-NA in mice, eliciting stress responses such as hyperactivation of SNS and HPA axis. A series of reactions resulted in the release of NA throughout the brain, significantly impacting attention, alertness, and short-term memory with the activation of memory-related neurons, such as DA neurons in the VTA, was suggested. Sustained activation of the NAc was also observed, leading to the hypothesis that stimulation of the FLs may be perceived as a visceral sensation (Fig. 8). However, the precise manner in which FLs' astringent stimuli are transmitted from the oral cavity and gastrointestinal tract to the brain remains unclear. The provision of a definitive response to this enquiry, and the elucidation of the role played by food sensory properties such as astringency in the preservation of homeostasis, is of significant importance for the maintenance and enhancement of human health.

## CRediT authorship contribution statement

Yasuyuki Fujii, Conceptualization, Data curation, Investigation, Visualization, Writing – original draft; Shu Taira, Methodology, Formal analysis; Keisuke Shinoda, Investigation, Visualization; Yuki Yamato, Investigation; Kazuki Sakata, Investigation; Orie Muta, Investigation; Yuta Osada, Investigation; Ashiyu Ono, Investigation,Visualization; Toshiya Matsushita, Investigation; Mizuki Azumi, Investigation; Hitomi Shikano, Investigation, Methodology; Keiko Abe, Writing – review and editing; Vittorio Calabrese: Writing – review and editing; Naomi Osakabe: Conceptualization, Funding acquisition, Supervision, Writing – review and editing.

All authors discussed the results and commented on the manuscript.

## Funding information

This work was supported by JSPS KAKENHI (Grant Number 23H02166).

## Declaration of competing interest

The authors declare that they have no known competing financial interests or personal relationships that could have appeared to influence the work reported in this paper.

## Data Availability

All data are available in the main text or supplementary file.
