# Supplementary material for: Astringent flavanol fires the locus-noradrenergic system, regulating neurobehavior and autonomic nerves
Source: Curr Res Food Sci. 2025 Sep 11;11:101195. doi: 10.1016/j.crfs.2025.101195 (PMC12482304; doi:10.1016/j.crfs.2025.101195)
Supplement: Multimedia component 1 [file mmc1.pdf]

Supplementary Information

Astringent flavanol fires the locus-noradrenergic system, regulating neurobehavior and autonomic nerves

Yasuyuki Fujii[1], Shu Taira[2], Keisuke Shinoda[3],Yuki Yamato[3], Kazuki Sakata[3], Orie Muta[3], Yuta Osada[3], Ashiyu Ono[3], Toshiya, Matsushita[4], Mizuki Azumi[3], Hitomi Shikano[2], Keiko Abe[5], Vittorio Calabrese[6], Naomi Osakabe[1,4,5*]

Affiliations

1. SIT Research Laboratories, Shibaura Institute of Technology.
2. Faculty of Food and Agricultural Sciences, Fukushima University
3. Functional Control Systems, Graduate School of Engineering and Science, Shibaura Institute of Technology
4. Department of Bio-science and Engineering, Shibaura Institute of Technology.
5. Department of Applied Biological Chemistry, Graduate School of Agricultural and Life Sciences, The University of Tokyo.
6. Department of Biomedical and Biotechnological Sciences, University of Catania.

*Complete address of corresponding author

Naomi Osakabe

Department of Bioscience and Engineering, Shibaura Institute of Technology, 307 Fukasaku, Minumaku, Saitama, 337-8570, Japan. Tel +81-48-720-6031, Fax +81-48-720-6011,

Email, nao-osa@shibaura-it.ac.jp

Materials and Methods

Materials

(-)-Epicatechin, dimer procyanidin B2, and B5, timer procyanidin C1 and tetramer cinnamtannnin A2 were purchased from Phyto Lab GmbH & Co. KG (Vestenbergsgreuth, ermany, Fig.1a). Caffeine and theobromine were purchased from Sigma-Aldrich (Sigma Aldrich, (St. Louis, MO). The isolation of flavanols (FLs) from cacao liquor was according to the method of Natsume et al. *(17)*. The Prussian Blue method measured total polyphenol concentration in FLs, using (-)- epicatechin as standard. The concentration of each flavanol or xanthin derivatives such as caffeine or theobromine was measured by the method of HPLC *(17)*. The composition of FLs derived from cocoa used in the experiment as follows; (-)-epicatechin (monomer Fig.1a left), 4.56%(W/W); (+)-catechin (monomer Fig.1a left), 6.43%; procyanidin B2(dimer, Fig.1a, right, N=0) , 3.93%; procyanidin C1 (trimer, Fig.1a, right, N=1), 2.36%; cinnamtannin A2 (tetramer, Fig.1a, right, N=2), 1.45 % as shown in Fig.1b.Xanthine derivatives (caffeine, theobromine) were below the detection limit.

Animals

10 weeks old male C57BL/6J mice were obtained from CLEA Japan, Inc. (Tokyo, Japan). During two weeks acclimation period, the animals were carefully handled for to reduce anxiety behaviors. Mice housed at room temperature (24-26 °C) under a 12-hour light/dark cycle (light cycle: 7:00-19:00, dark cycle: 19:00-7:00) with free access to water and food. The solid diet (MF) for laboratory animals was obtained from Oriental Yeast Co., Ltd. (Tokyo, Japan). The study was conducted following the Code of Ethics in compliance with the Declaration of Helsinki, and the protocol was approved by the Animal Experimentation Committee of Shibaura Institute of Technology (approval number: AEA 22007). In addition, all animals were humanely raised according to the ARRIVE guidelines of this period.

Open filed test for observation of spontaneous behavior

We observed the effects of a single oral dose of FLs on the spontaneous behavior of mice in the open-field test. Mice were orally administrated with distilled water (D.W.), or FLs 25 mg/kg body weight. The open fields are composed of four transparent acrylic chambers aligned horizontally or vertically. Mice were immediately placed in the center of an arena (40 x 40 x 40 cm), and their behavior was recorded for 120 min. The traveled distance was analyzed from the data, which were exported as a 30-min mp4 at 25 fps, corrected for tilt and brightness on filming using Premire Pro (Adobe Inc., California, USA), with a modification of the automatic tracking system developed by Zhang et al.*(68).* All video files were processed by a MATLAB script running on a PC. The mouse activity code was designed to detect black mice such as C57BL/6J in a light arena.

Novel object recognition test

Novel object recognition test was performed according to the method of Ligar et al. with minor modifications as shown in Fig.2a *(18).* After two weeks of acclimation, mice were divided into the D.W.-treated group and the FLs 25mg/kg-treated group (n=8 each). A mouse given D.W. or FLs by gavage was placed in the center of an arena made from transparent acrylic (40 x 40 x 40 cm) and allowed to adapt for one hour. Two identical objects were placed in opposite quadrants of the arena (i.e., the northwest and southeast corners) and the mice were allowed 10 min to explore them freely. One hour later, one of the objects was replaced by a novel object of a different shape, and material, and the mice were allowed to explore the arena for 8 min to perform a novel object recognition test. The term "exploration" was defined as behavior exhibited by mice whereby the mouse's noise is directed towards an object and within a distance of 2 cm from the object, with active vibrissae sweeping or sniffing. The tally did not include the time spent sitting on the object without indicating active exploration. The decision exploring or not exploring was analyzed from the data, which were exported as a 30-minute mp4 at 25 fps,

corrected for tilt and brightness on filming using Premire Pro (Adobe Inc., California, USA), with a modification of the automatic tracking system developed by Zhang et al. *(68).* All video files are processed by a MATLAB script running on a PC. The mouse activity code was designed to detect black mice such as C57BL/6J in a light arena. To assess working memory, the discrimination index (DI) was calculated as the total exploration time to the novel object divided by the total exploration time, as previously described in a paper by Leger et al. *(18)* as follows. [(time spent exploring novel object) − (time exploring familiar object)]/[(time spent exploring novel object)+(time exploring familiar object)].

Quantification of urinary CA excretion using HPLC

It has been suggested that brief periods of social isolation in metabolic cages can markedly alter sympathetic nervous system activity in mice *(62).* In our preceding research, we demonstrated that co-housing two mice in metabolic cages markedly reduced the stress response associated with single housing (19). Consequently, in the present study, we employed this approach to examine the sympathetic nervous system activity of FLs. Following a 48-hour acclimation period, urine was collected for 24 hours using a tube containing 20 μl of 2.5 mol/L HCl following oral administration of test chemicals. Oral administration of DW or FLs was performed between 10:00 and 11:00.

Noradrenaline (NA) and adrenaline (AD) was purchased from Sigma–Aldrich. The urine was neutralized and heated for 10 min following incubation with 500 U/ml of the enzyme sulfatase from Helix pomatia Type H-2 (Sigma–Aldrich) for one hour at 37°C. Following this, isoprenaline (Sigma–Aldrich) was added as the internal standard, after which CA was purified using Monospin PBA® (GL Sciences, Tokyo, Japan). The HPLC system (Prominance HPLC System Shimazu Corporation, Kyoto, Japan) comprised a quaternary pump with a vacuum degasser, a thermostatted column compartment, and an autosampler equipped with an electrochemical detector (ECD 700 S; Eicom Corporation, Kyoto, Japan). A reverse-phase

column (Inertsil ODS-4, 250 × 3.0 mm ID, 5 µm; GL Sciences) was utilized, with the column temperature maintained at 35°C. The HPLC mobile phase comprised 24 mM acetate-citrate buffer (pH 3.5, -CH3CN, 100/14.1 v/v). The mobile phase flow rate was 0.3 ml/min, with an injection volume of 20 µl. Eluents were detected and analyzed at 500 mV. The excretion of CA was expressed as a ratio with the urinary creatinine concentration, which was measured using Laboassay creatinine (FUJIFILM Wako Pure Chemical Corporation).

Observation of c-fos and CRH dynamics using ISH

Mice were gavaged with either distilled water (DW) or 25 mg/kg flavan 3-ols (FLs) and then decapitated 15, 30 or 60 minutes later. The excised brains were immediately frozen and coronally sectioned (8 µm thick) using a cryostat (Leica, Wetzlar, Germany). The sections of fresh-frozen brains were prepared with a cryostat and thaw-mounted on glass slides (Matsunami Glass Ind. Ltd., Osaka, Japan). *In situ* hybridization was carried out to evaluate c-fos and CRH mRNA expression with the RNAscope® Multiprex Assay (Advanced Cell Diagnostics, CA, USA). An ImmEdge™ pen (H-4000, Vector Laboratories Inc., California, USA) was used to create a barrier around the sections. The barriers were dried completely at room temperature. The samples were treated with hydrogen peroxide (RNAscope® H_2O_2 and Protease Plus Reagents, Advanced Cell Diagnostics, CA, USA) for 10 min at room temperature, and washed twice in distilled water. The antigens were activated with RNAscope® Target Retrieval Reagents (Advanced Cell Diagnostics) for 15 min, washed immediately in distilled water, and dehydrated in ethanol for 1 min. The sections were treated with Protease Plus Reagents for 30 min at 40 °C in the HybEZ™ OVEN (Advanced Cell Diagnostics); then the sections were washed twice in distilled water. Next, the RNA probe solution was mixed (1:50 dilution) with the target RNA probes: Mm-Fos (316921, Advanced Cell Diagnostics) and Mm-Crh-C2 (316091-C2, Advanced Cell Diagnostics). The sections were incubated with the probes for 2 h at 40 °C, then washed twice in wash buffer (Advanced Cell Diagnostics, CA, USA). Next, the

sections were treated serially with AMP1 for 30 min(Advanced Cell Diagnostics), AMP2 for 15 min(Advanced Cell Diagnostics), AMP3 for 30 min(Advanced Cell Diagnostics), AMP4 for 15 min(Advanced Cell Diagnostics) at 40 °C, and AMP5 for 30 min(Advanced Cell Diagnostics), and AMP6 for 15 min (Advanced Cell Diagnostics) at room temperature. The sections were washed twice in wash buffer for 2 min and treated with the red conjugates (60:1), Fast red A and Fast red B, for 10 min at room temperature. After washing in wash buffer, the sections were treated successively with AMP7 for 15 min(Advanced Cell Diagnostics), AMP8 for 30 min(Advanced Cell Diagnostics) at 40 °C, and AMP9 for 30 min(Advanced Cell Diagnostics), and AMP10 for 15 min (Advanced Cell Diagnostics) at room temperature. Next, the sections were treated with green conjugates (50:1) as Fast green A and Fast green B (Advanced Cell Diagnostics), for 10 min at room temperature. The sections were rinsed twice in distilled water. Finally, the sections were immersed in 50% Gill hematoxylin (GHS116-500ML, Sigma Aldrich, St-Louis, USA) for 30 s and washed twice immediately in water. After drying completely in a dry oven (MIR-162-PJ, Panasonic, Osaka, Japan) at 60 °C, the sections were covered with Vector mount™ permanent mounting medium (H-5000, Vector Laboratories Inc., California, USA).

Observation of neurotransmitter location

Mice were gavaged with either distilled water (DW) or 25 mg/kg flavan 3-ols (FLs) and then decapitated immediately after, 15 or 60 min later. The excised brains were immediately frozen and sectioned (8 μm thick) using a cryostat (Leica, Wetzlar, Germany). The sections of fresh-frozen brains were prepared with a cryostat and thaw-mounted on conductive indium-tin-oxide-coated glass slides (Matsunami Glass Ind. Ltd., Osaka, Japan). A pyrylium-based derivatization method was applied for the tissue localization imaging of NA, its precursors L-dopa and dopamine (DA), and its metabolite normetanephrine (NMET) according to the method by previous publication *(70).* In brief, a solution of TMPy (4.8 mg/200 μL) (Taiyo Nippon Sanso

Co., Tokyo, Japan) was applied to brain sections using an airbrush (Procon Boy FWA Platinum 0.2-mm caliber airbrush, Mr. Hobby, Tokyo, Japan). To enhance the reaction efficiency of TMPy on sections, the TMPy-sprayed sections were placed into a dedicated container and allowed to react at 60 °C for 10 min. The container contained two channels in the central partition, to wick moisture from the wet filter paper region to the sample section region. The filter paper was soaked with 1 mL methanol/water (70/30 volume/volume) and placed next to the section inside the container, which was then completely sealed to maintain humidity levels. The TMPy-labeled brain sections were sprayed with matrix (α-cyano-4-hydroxycinnamic acid-methanol/water/TFA = 70/29.9/0.1 volume/volume) using an automated pneumatic sprayer (TM-Sprayer, HTX Tech., Chapel Hill, NC, USA). Ten passes were sprayed according to the following conditions: flow rate, 120 μL/min; airflow, 10 psi; nozzle speed, 1100 mm/min. Each section was scanned to detect the laser spot area, and laser spot areas (200 shots) were detected with a spot-to-spot center distance of 120 μm using MALDI-Q-TOF MSI (rapifleX®, Bruker Daltonics Bremen, GmbH). The section surface was irradiated with yttrium aluminum garnet laser shots in the positive ion detection mode. The laser power was optimized to minimize the in-source decay of targets. Signals between m/z 200-800 were collected. Obtained mass spectrometry spectra were reconstructed to produce mass spectrometry images using Scils Lab software (Bruker Daltonics). Optical images of brain sections were obtained using a scanner (GT-X830, Epson, Tokyo, Japan) followed by MALDI-TOF MS of the sections. The detected masses of TMPy-labeled standard chemicals increased by 105.0 Da compared with the original mass, TMPy-l L-dopa, m/z 302.1; TMPy-l DA, m/z 258.1; TMPy-l NA, m/z 274.1 ; TMPy-l NMET, m/z 288.2. Tandem mass spectrometry confirmed the fragmentation ions of TMPy from the standard sample. A fragmented ion of the pyridine ring moiety was regularly cleaved and observed for all TMPy-modified target molecules.Tandem mass spectrometry confirmed the fragmentation ions of TMPy from the standard sample.

Observation of noradrenaline dynamics using ISH

The mRNA expression of tyrosine hydroxylase (TH), dopamine β-Hydroxylase (DBH), and vesicular monoamine transporter (VMAT) 2 was observed by ISH using consecutive sections from the sections used in the MS imaging analysis. *In situ* hybridization was carried out to evaluate TH, DBH and VMAT mRNA expression with the RNAscope® Multiprex Assay (Advanced Cell Diagnostics) as described above. The target RNA probes were follows; TH (Probe-Mm-Th-C4, 317621-C4, Advanced Cell Diagnostics), DBH (Probe-Mm-Dbh-C3, 400921-C3, Advanced Cell Diagnostics), VMAT2(Probe Mm-Slc18a2, 25331, Advanced Cell Diagnostics).

Fluorescence microscopy and quantification

All image analyses were performed by experimenters blinded to the experimental conditions. ISH image was taken using a fluorescence digital microscope (BZ-X800, KEYENCE Corp., Osaka, Japan). Cell counting of c-fos and CRH were performed throughout the extend of PVN. Each expression was quantified using NIH Image J software ver. 1.52 (http://rsb.info.nih.gov/ij/index.html).

Statistical Analysis

The sample size was determined from the results of a preliminary experiment using a power test with a significance level of 0.05 and power 0.9. All quantitative assessments were carried out under blinded manner. No data were excluded from the analyses. All data were expressed as mean ± standard deviation. All data were analyzed using GraphPad Prism 10.2.3 (San Diego, CA, USA). The normal distribution of the sample was tested using the Shapiro-Wilk test. Comparisons between the two groups were made using the Student's t test or the Mann-Whitney test. For statistics on urinary CAs, the Kruskal-Wallis test was performed followed by Dunn's multiple comparisons test. Otherwise, two-way ANOVA followed by the Tukey’s multiple

comparisons test as post hoc test was used for multiple comparisons. The significance levels were defined at # $p<0.1$, * $p<0.05$, ** $p<0.01$, *** $p<0.001$.

Supplemental results

Results of spontaneous behavior observation

The subsequent behavioral trajectories of all mice in the arena over a 120-min period were shown in SFig.1.

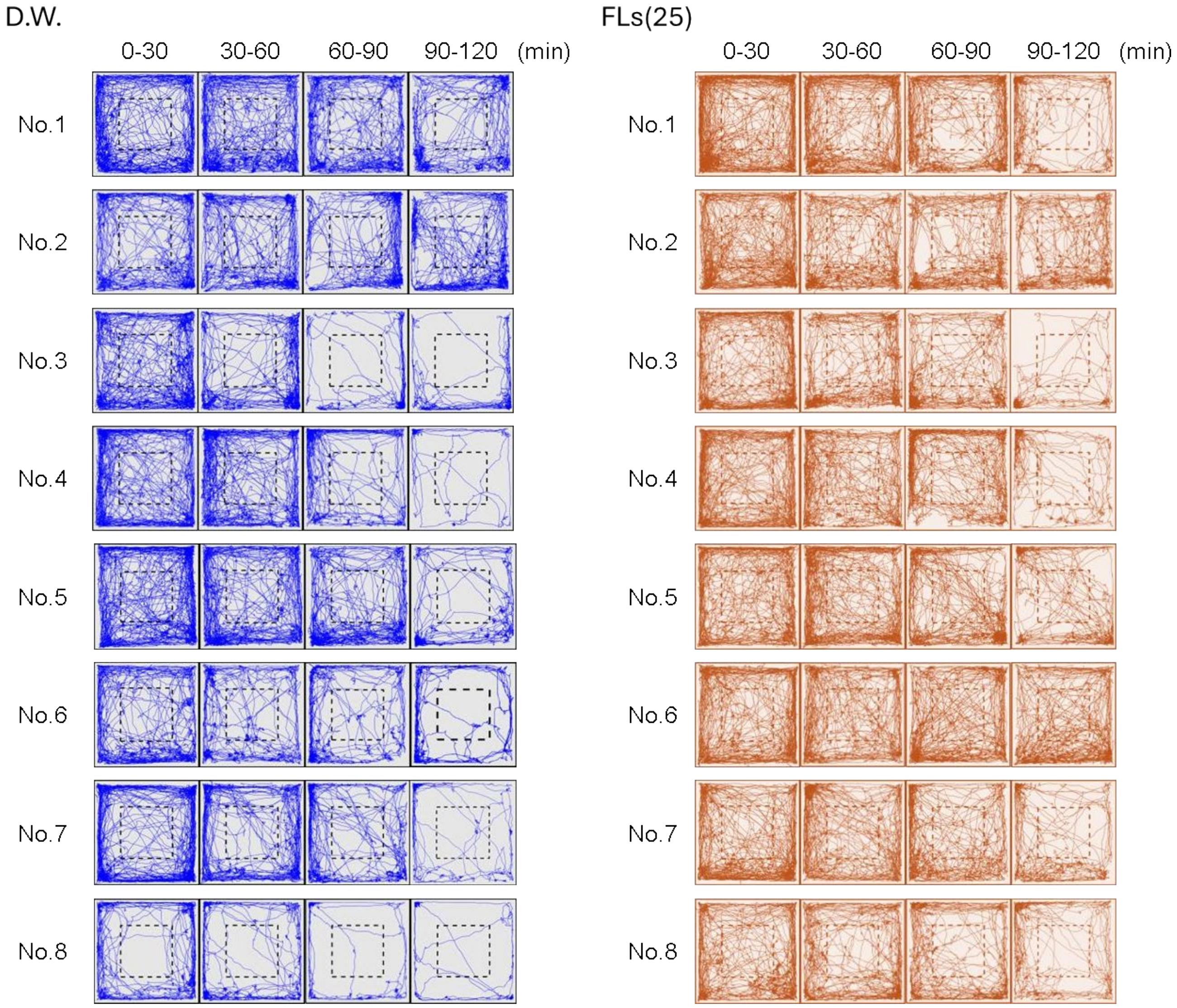

SFig.1 Behavioral trajectories in the arena for all mice for 120 min following a single oral administration of distilled water or 25mg/kg flavanol (FLs).

Results of novel object recognition test

The overall results were shown in Fig. 2. Additionally, typical mouse behavior was also shown in the supplemental video.

Impact of FLs on HPA axis as observed by mRNA expression of c-fos and CRH in PVN

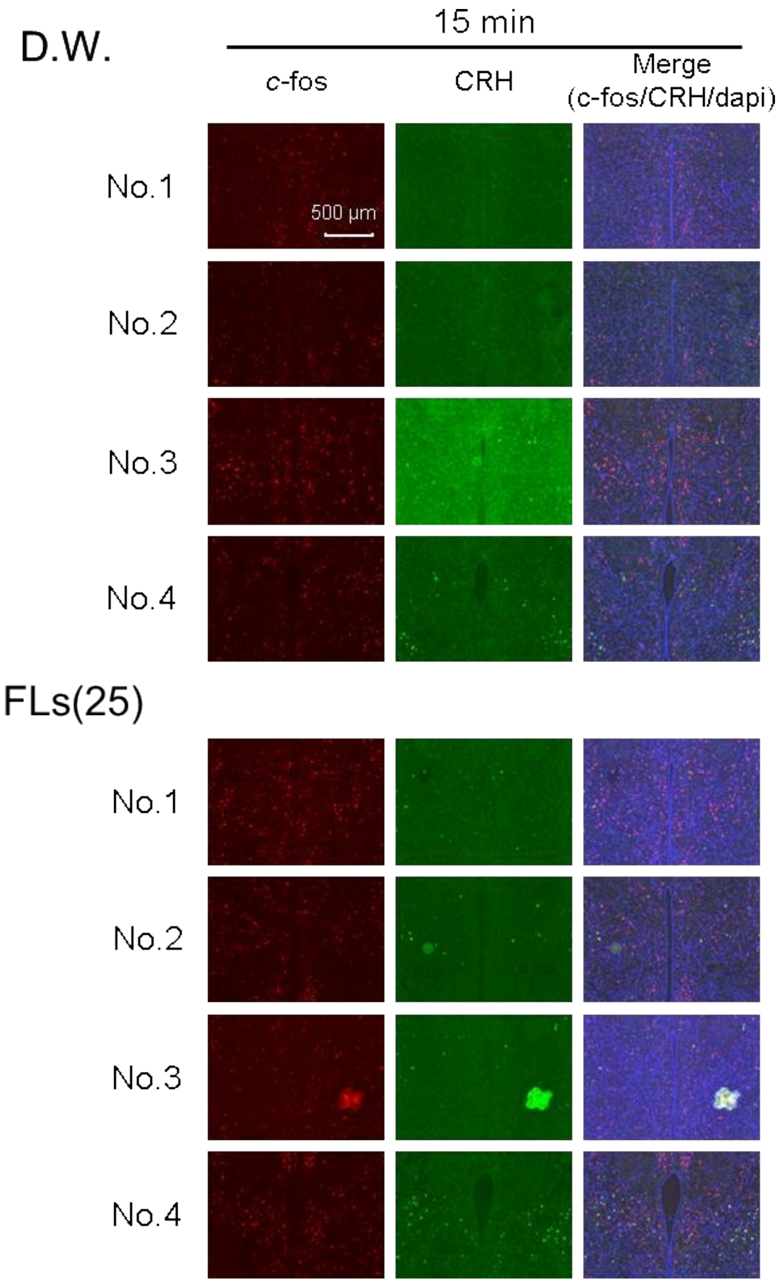

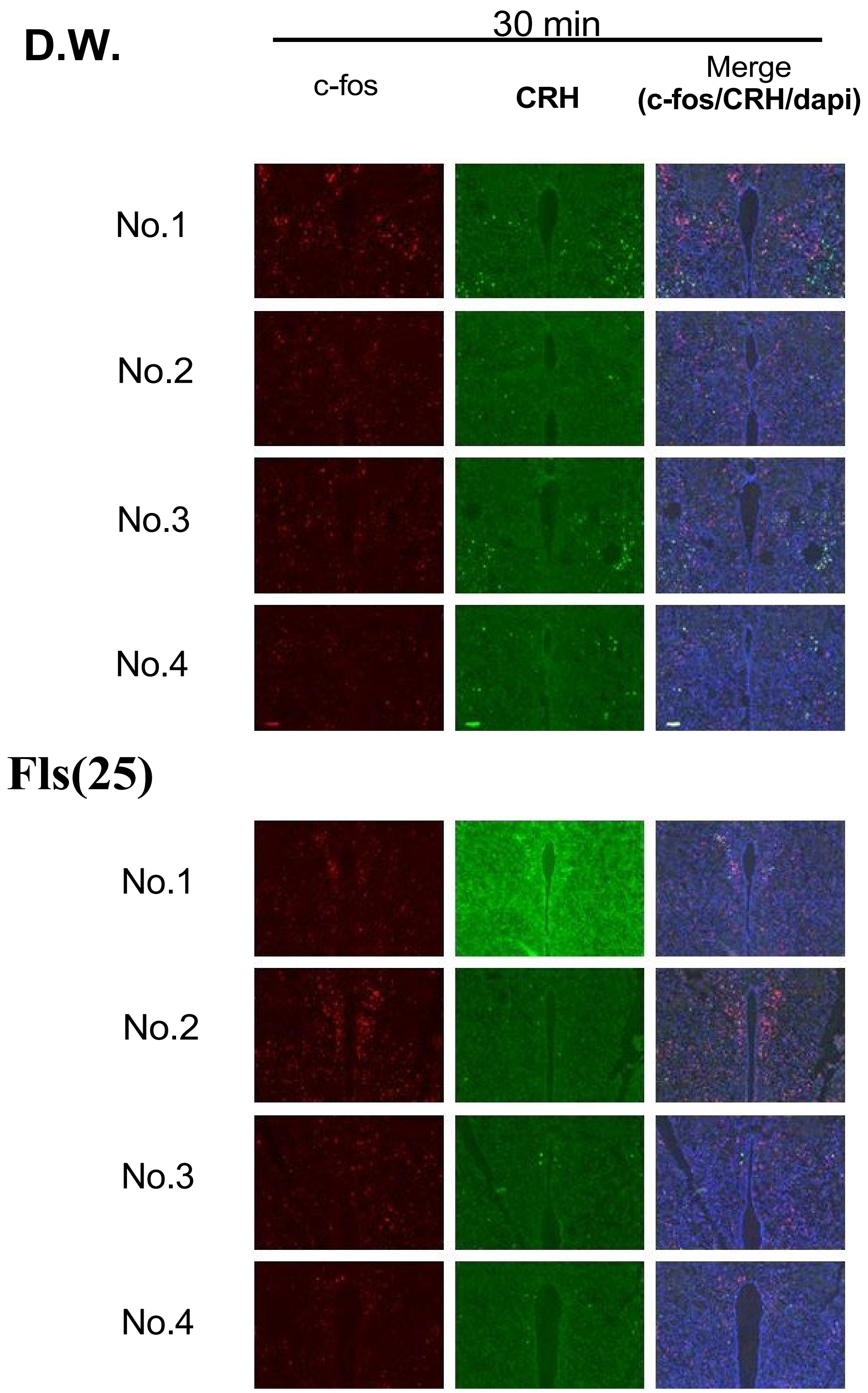
D.W.
30 min
c-fos
CRH
Merge
(c-fos/CRH/dapi)
No.1
No.2
No.3
No.4
Fls(25)
No.1
No.2
No.3
No.4

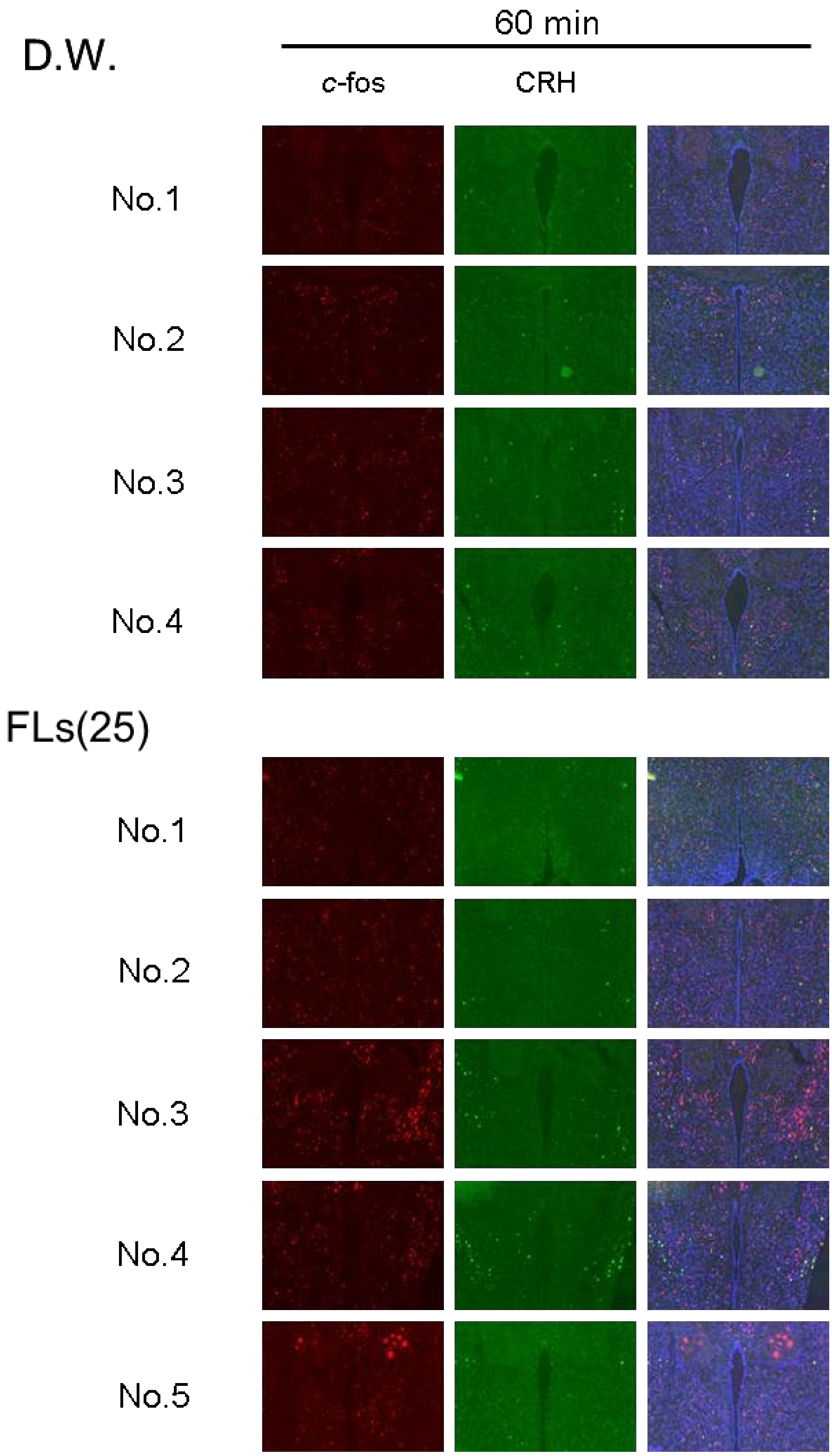

SFig.2 The results of observing the mRNA expression of cfos and the stress hormone CRH in the hypothalamic PVN of all mice using the ISH method.

The change of L-dopa, dopamine, noradrenaline and its dynamics in metabolite norethanephrine (NMET) in whole mouse brain observed by the method of MS imaging

SFig.3 showed the distribution and respective intensities of noradorenaline, its precursors L-dopa and dopamine, and its metabolite norethanephrine detected in sagittal sections of mouse brain immediately after, 15 or 60 min after a single dose of D.W. or 25 mg/kg FL.

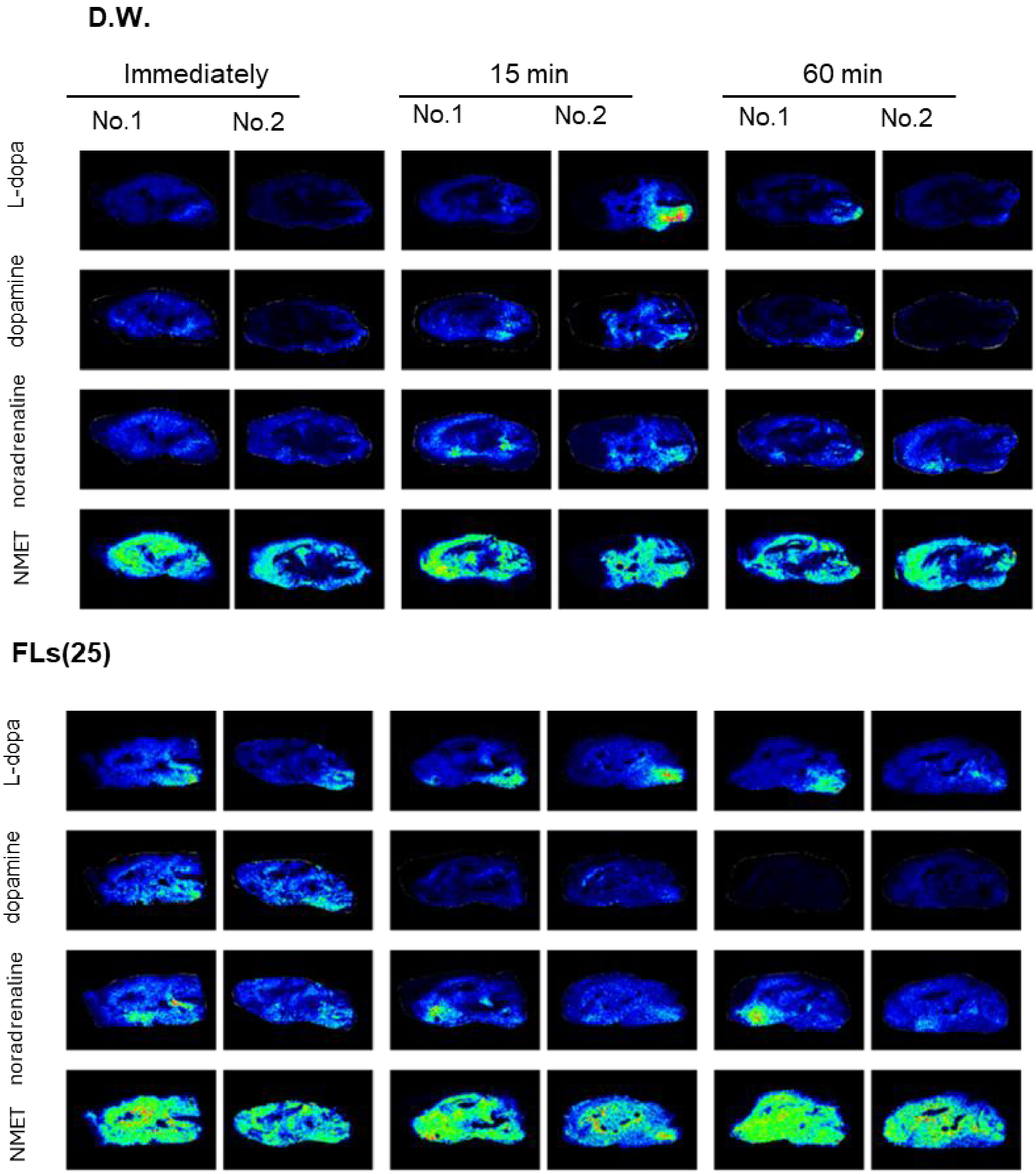

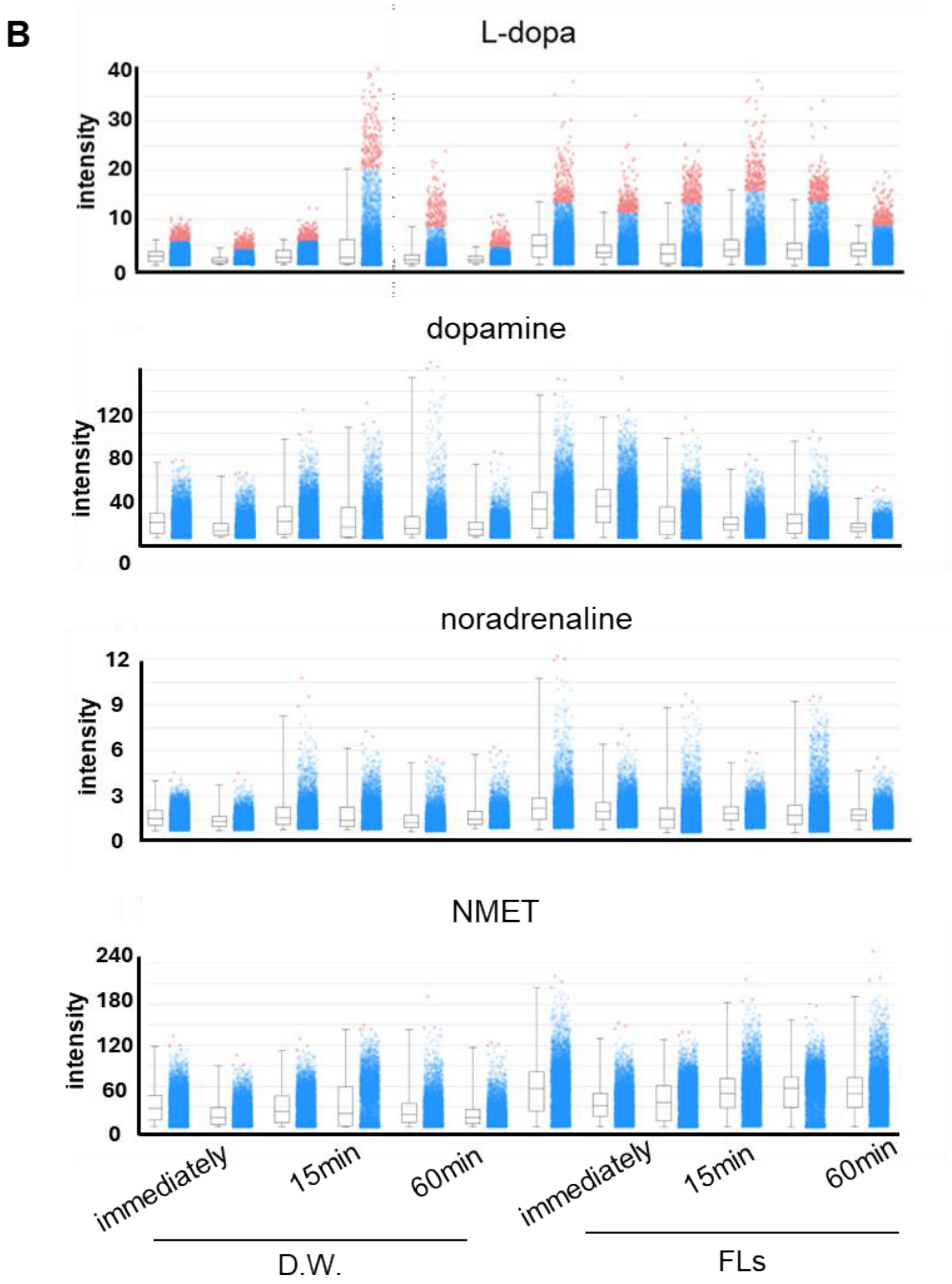

SFig.3 The intensities of L-dopa, dopamine, noradrenaline and normetanephrine (NMET) immediately, 15 min, and 60 min after a single oral administration of flavanols (FLs) in mice whole brain.A Representative MS images of L-dopa (top), dopamine (DA, second from top), noradrenaline (NA, third from top), and normetanephrine (NMET, bottom). B Relative intensity of L-dopa, DA, NA, and NMET for each animal.

Intensity of noradrenaline observed in mice LC, LPO, and NAc by the method of MS imaging

SFig.4 showed the distribution and respective intensities of noradorenaline in LC, LPO, and ACB of mouse brain immediately after, 15 or 60 min after a single dose of D.W. or 25 mg/kg FL.

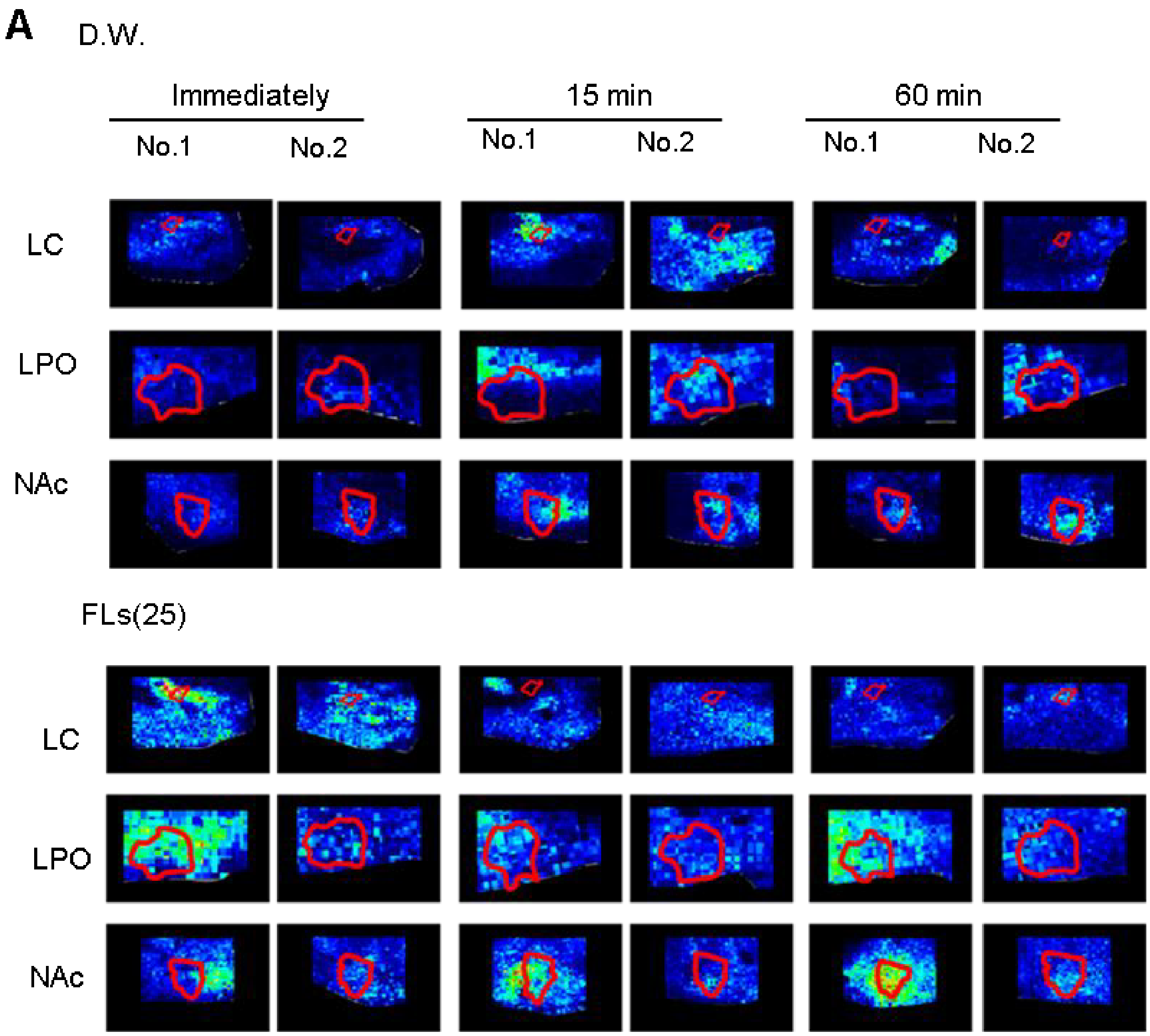

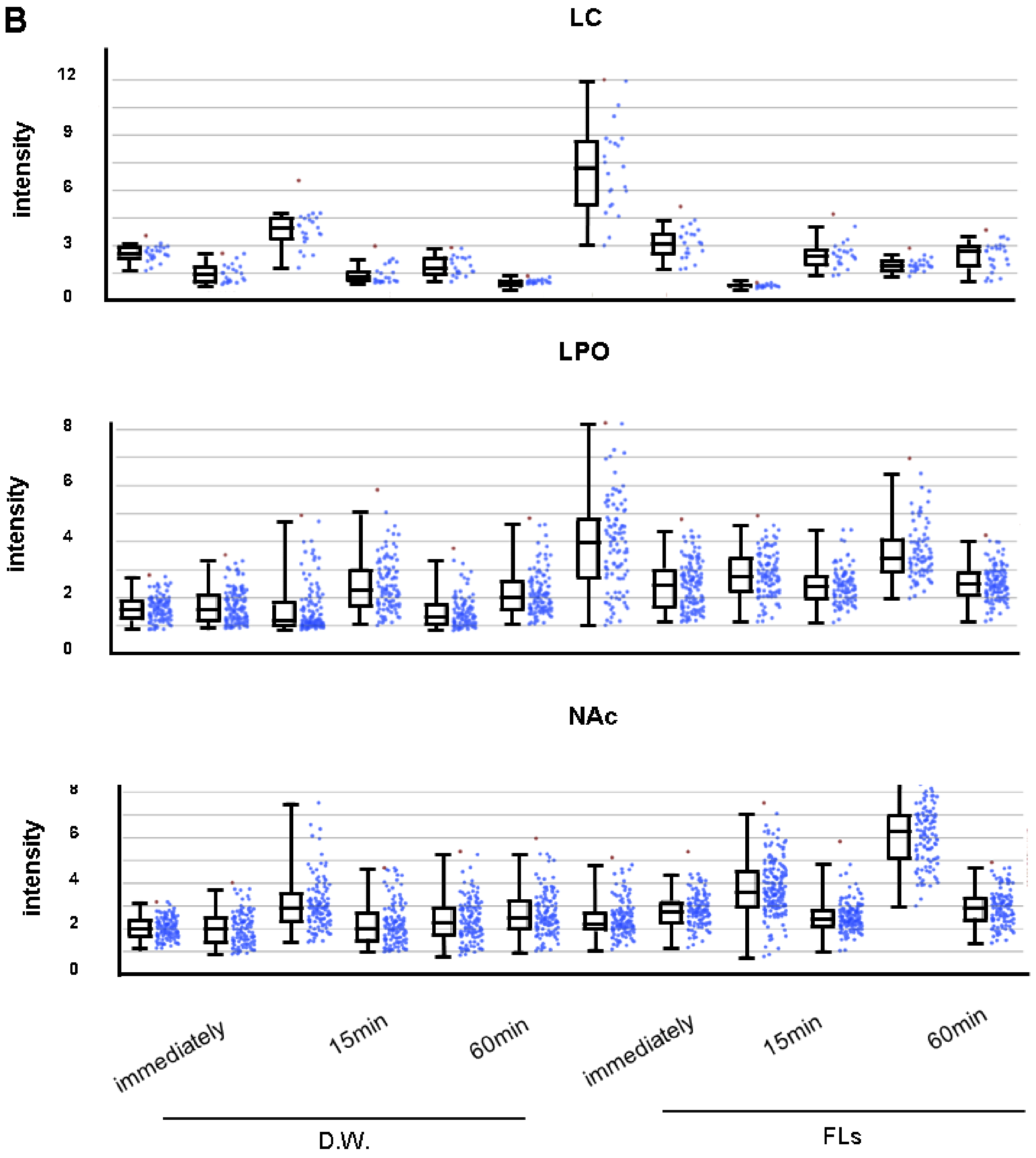

SFig.4 The intensities of noradrenaline immediately, 15 min, and 60 min after a single oral administration of flavonols (FLs) in locus coeruleus (LC), lateral preoptic area (LPO), and nucleus accumbens (ACB) . A Representative MS images and relative intensity of noradrenaline LC (top), LPO (middle) and ACB(bottom) . B Relative intensity of noradrenalin in LC, LPO and ACB.

The change of mRNA expression catecholamine synthesis enzymes and its transporter in

the brain observed by the method of ISH

SFig.5 showed the change of mRNA expressions in tyrosine hydroxylase (TH), dopamine β-hydroxylase (DBH), and vesicular monoamine transporter (VMAT)2 in LC and ventral tegmental area (VTA).

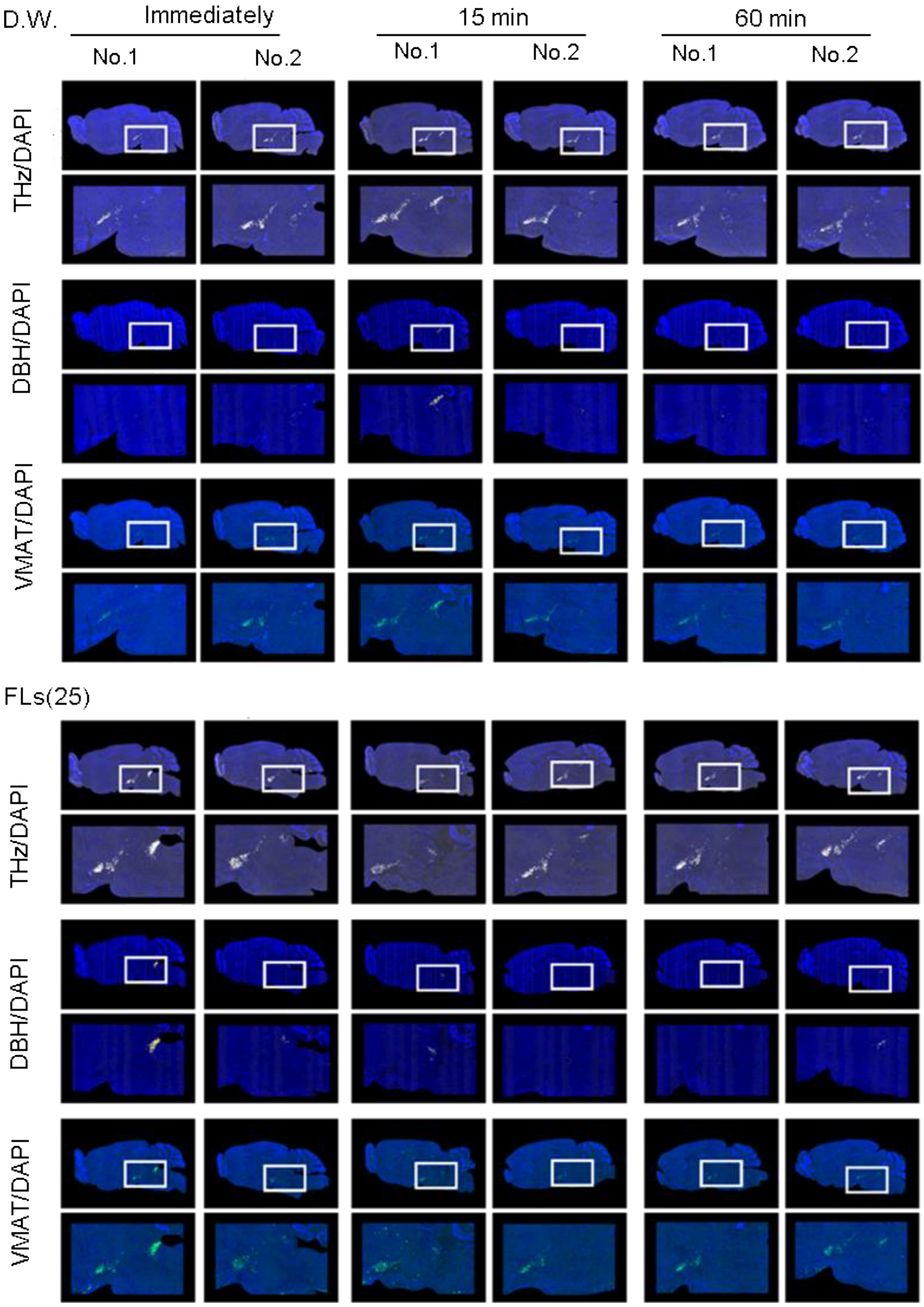

SFig.5 The change of mRNA expression in TH, DBH and VMAT 2 in LC or VTA of all mice following oral administration of D.W or FLs.

Supplemental Movie

Svideo 1
The behavioral trajectory of mice in the NOT test after administration of distilled water(D.W.)
A movie of mouse behavioral trajectory in the NOT test after administration of D.W shown in Fig. 2B(left)

Svideo2
The Behavioral trajectory of mice in the NOT test after administration of Flavanols (FLs)
A movie of mouse behavioral trajectory in the NOT test after administration of FLs shown in Fig. 2B (right)

Figure 1e
Traveled distance every 10min (m)

Time (min)	DW								FLs(25)							
	1	2	3	4	5	6	7	8	1	2	3	4	5	6	7	8
10	30.242	25.136	32.496	21.33	33.482	23.888	31.231	19.978	50.873	40.752	33.037	32.464	29.815	38.939	31.59	32.113
20	28.593	18.44	25.349	30.256	34.886	21.132	31.697	18.07	37.375	28.709	27.362	28.009	36.053	26.066	26.354	19.916
30	28.204	22.126	23.874	29.893	25.692	23.104	26.032	13.252	28.592	27.067	29.955	32.524	22.323	30.876	32.923	26.149
40	27.531	20.206	23.47	27.32	30.906	15.338	20.531	15.497	32.88	22.769	26.835	24.901	34.7	24.488	28.668	25.886
50	25.69	21.865	19.71	27.91	27.68	17.074	26.892	13.377	25.39	26.741	22.664	25.384	31.061	24.855	22.552	22.17
60	27.987	21.199	13.45	21.686	24.311	16.322	20.943	12.125	25.39	26.741	22.664	25.384	31.061	24.855	22.552	22.17
70	24.762	19.9	12.166	26.156	28.819	13.692	21.263	9.37	30.494	21.355	21.248	25.127	30.882	31.328	25.639	24.115
80	21.245	18.485	8.119	8.25	24.925	14.713	18.79	4.696	29.257	25.048	22.718	22.611	24.825	28.363	23.971	23.086
90	21.914	17.652	6.722	15.005	15.873	16.474	15.069	8.741	18.909	20.048	18.876	25.51	21.948	29.311	18.643	14.844
100	17.953	22.145	8.446	8.689	13.923	8.261	15.735	9.78	24.493	21.861	13.693	15.254	17.613	28.665	14.718	19.175
110	18.65	20.872	13.939	6.765	17.653	17.521	7.527	4.74	13.385	19.687	8.446	10.695	18.832	28.399	18.886	14.129
120	14.979	20.567	3.048	1.082	6.883	5.177	5.64	1.292	10.659	19.945	6.715	10.471	14.845	25.203	11.76	13.441

Figure 1f
Total travel distance, accumulation (m)

Time (min)	DW								FLs(25)							
	1	2	3	4	5	6	7	8	1	2	3	4	5	6	7	8
10	30.24198	25.13628	32.49636	21.3299	33.48205	23.88843	31.23129	19.97831	50.87322	40.75152	33.03747	32.46392	29.81543	38.93866	31.59029	32.11329
20	58.83535	43.57591	57.84494	51.58607	68.36847	45.02	62.92833	38.04819	88.24788	69.46095	60.39898	60.47266	65.86809	65.00508	57.94463	52.02955
30	87.0389	65.70235	81.7189	81.47881	94.06051	68.12426	88.9603	51.29981	116.84	96.52787	90.35436	92.99688	88.19071	95.88076	90.86782	78.17817
40	114.5695	85.90824	105.1884	108.799	124.9669	83.4625	109.4913	66.79658	149.7198	119.2972	117.1898	117.8977	122.8903	120.3692	119.5363	104.064
50	140.2593	107.7734	124.8986	136.7089	152.6471	100.5361	136.3836	80.17357	175.1098	146.0387	139.8534	143.2817	153.951	145.2241	142.0881	126.2343
60	168.2465	128.9724	138.349	158.3953	176.958	116.8577	157.3263	92.29818	200.4998	172.7801	162.5169	168.6657	185.0117	170.0789	164.6399	148.4045
70	193.0081	148.8727	150.5152	184.5518	205.7774	130.5493	178.5889	101.6677	230.9939	194.1348	183.7648	193.793	215.8936	201.4064	190.2786	172.519
80	214.2535	167.3579	158.6343	192.8019	230.7019	145.2621	197.3787	106.3636	260.2511	219.1829	206.4824	216.4045	240.7186	229.7693	214.25	195.6049
90	236.1673	185.0094	165.356	207.8071	246.5752	161.7359	212.4477	115.1046	279.1601	239.2305	225.3588	241.9149	262.6667	259.0801	232.8927	210.4492
100	254.1199	207.1541	173.8022	216.496	260.4986	169.9966	228.1828	124.8848	303.6528	261.0918	239.0521	257.1693	280.2798	287.745	247.6111	229.6242
110	272.7702	228.0264	187.7411	223.261	278.1517	187.5174	235.7097	129.6252	317.0382	280.7791	247.4979	267.8647	299.1119	316.144	266.4975	243.7528
120	287.7489	248.5937	190.7895	224.343	285.035	192.6939	241.3498	130.9168	327.6974	300.724	254.2131	278.3356	313.9565	341.3471	278.2576	257.1939

	**Figure 1g** Total travel distance (m)		**Figure 1h** Total spent time in center area (s		**Figure 1i** Grooming(number)		**Figure 1j** Rearing(number)	
	DW	FLs(25)	DW	FLs(25)	DW	FLs(25)	DW	FLs(25)
1	287.7489	327.6974	311.16	379.12	52	69	383	680
2	248.5937	300.724	407.76	842.2	42	46	432	584
3	190.7895	254.2131	143.56	358.44	33	63	290	479
4	224.343	278.3356	296	453.68	22	59	373	470
5	285.035	313.9565	323.4	527.84	27	62	468	734
6	192.6939	341.3471	359.64	817.72	41	46	363	756
7	241.3498	278.2576	256.92	497.64	41	56	418	606
8	130.9168	257.1939	38.44	343.64	41	40	200	524

Figure 2c
Exploration time (sec)

Figure 2d
DI

ID	DW		FLs(25)		DW	FLs(25)
-	Familiar	Novel	Familiar	Novel		
1	14.875	14.29	9.995	18.21	0.489971	0.64563
2	7.275	11.825	10.36	22.275	0.61911	0.682549
3	11.14	7.8	5.335	19.835	0.411827	0.788041
4	9.925	11.235	8.715	25.375	0.530955	0.744353
5	12.58	12.335	10.815	19.78	0.495083	0.646511
6	11.105	8.06	9.05	20.715	0.420558	0.695952
7	11.51	11.35	10.525	11.135	0.4965	0.514081
8	9.11	11.935	8.885	20.125	0.567118	0.693726

Figure 3b
Urine adrenaline (ng/µg Cre)

Figure 3c
Urine noradrenaline (ng/µg Cre)

Figure 3d
Urine noradrenaline + adrenaline (ng/µg Cre)

ID	3b DW	3b FLs(25)	3b FLs(50)	3c DW	3c FLs(25)	3c FLs(50)	3d DW	3d FLs(25)	3d FLs(50)
1	0.069	0.173	0.28	0.069	0.173	0.28	0.37	0.383	0.843
2	0.108	0.165	0.163	0.108	0.165	0.163	0.356	0.529	0.613
3	0.145	0.122	0.274	0.145	0.122	0.274	0.416	0.521	0.781
4	0.162	0.145	0.338	0.162	0.145	0.338	0.555	0.473	0.731
5	0.06	0.213	0.322	0.06	0.213	0.322	0.321	0.559	0.813
6	0.142	0.051	0.283	0.142	0.051	0.283	0.275	0.398	0.847
7	0.116	0.277	0.277	0.116	0.277	0.277	0.406	0.701	0.791
8	0.15	0.194	0.377	0.15	0.194	0.377	0.602	0.34	0.874
9	0.175			0.175			0.585		
10	0.184			0.184			0.52		
11	0.185			0.185			0.587		
12	0.174			0.174			0.506		
13	0.241			0.241			0.723		
14	0.228			0.228			0.674		
15	0.245			0.245			0.569		

Figure 4c
c-fos mRNA labeled cells

Time (min)	DW				FLs(25)				
ID	1	2	3	4	1	2	3	4	5
15	16	7	26	13	25	21	17	18	
30	23	12	14	10	35	50	15	9	
60	5	23	15	7	9	12	9	6	13

Figure 4d
CRH mRNA labeled cells

Time (min)	DW				FLs(25)				
ID	1	2	3	4	1	2	3	4	5
15	8	9	11	5	13	3	10	1	
30	4	10	0	2	37	24	7	30	
60	6	16	7	3	13	1	0	3	9
